# Osteohistology of a Triassic dinosaur population reveals highly variable growth trajectories typified early dinosaur ontogeny

**DOI:** 10.1038/s41598-022-22216-x

**Published:** 2022-10-15

**Authors:** Daniel E. Barta, Christopher T. Griffin, Mark A. Norell

**Affiliations:** 1 Present Address: Department of Anatomy and Cell Biology, Oklahoma State University College of Osteopathic Medicine at the Cherokee Nation, Tahlequah, OK USA; 2 Richard Gilder Graduate School and Division of Paleontology, American Museum of Natural History, New York, NY USA; 3 Department of Earth and Planetary Sciences, Yale University, New Haven, CT USA; 4 Yale Peabody Museum of Natural History, Yale University, New Haven, CT USA

**Keywords:** Palaeontology, Palaeontology

## Abstract

Intraspecific variation in growth trajectories provides a fundamental source of variation upon which natural selection acts. Recent work hints that early dinosaurs possessed elevated levels of such variation compared to other archosaurs, but comprehensive data uniting body size, bone histology, and morphological variation from a stratigraphically constrained early dinosaur population are needed to test this hypothesis. The Triassic theropod *Coelophysis bauri*, known from a bonebed preserving a single population of coeval individuals, provides an exceptional system to assess whether highly variable growth patterns were present near the origin of Dinosauria. Twenty-four histologically sampled individuals were less than a year to at least four years old and confirm the right-skewed age distribution of the *Coelophysis* assemblage. Poor correlations among size, age, and morphological maturity strongly support the presence of unique, highly variable growth trajectories in early dinosaurs relative to coeval archosaurs and their living kin.

## Introduction

Intraspecific variation is the fuel that powers evolution by natural selection, and the life history of an organism is a major determinant of its body size, morphology, ecology, and ultimate reproductive success^1^. Therefore, intraspecific variation in ontogenetic trajectories—the changes undergone during life history—is evolutionarily critical because variation in growth timing, rate, duration, and body size at maturity can lead to differential survival, fitness, and fecundity of the members of a population^2–4^. Levels of intraspecific variation in postnatal ontogeny differ among vertebrate groups, with a clear difference between the two clades of living archosaurs, Crocodylia and Aves. Crocodylians exhibit a relatively high degree of morphological, histological, and body size variation during growth^5–8^. Indeed, similar levels of variation are widespread among non-avian and non-mammalian amniotes^9–13^ and may be plesiomorphic for Tetrapoda as a whole^14,15^. In contrast, some birds are thought to have lower levels of such growth variation, potentially as a consequence of their sustained rapid growth^6,15,16^ and ability to compensate for periods of short-term nutrient limitation with faster growth, which may return a bird to a normal growth trajectory before it reaches maturity^3^. The difference between extant crocodilians and birds suggests that a transition or transitions to reduced ontogenetic variation occurred within avian-line archosaurs. To better contextualize the ancestral degree of ontogenetic variation within avian-line archosaurs, its reduction in some taxa, and the biological processes underscoring this disparity in variation, it is necessary to examine multiple types of ontogenetic data from their extinct relatives, especially non-avian dinosaurs.

The earliest dinosaurs have been suggested to have extremely high levels of intraspecific variation in growth pattern and ontogenetic trajectory—potentially higher than even living crocodylians. Osteohistology of the Triassic sauropodomorph *Plateosaurus* revealed high intraspecific variation in body size for a given ontogenetic age, which was interpreted as indicative of developmental plasticity^17^. Similar variation has recently been reported in the osteohistology of another early sauropodomorph, *Massospondylus*^14^, although in both studies the relatively large sample was taken from multiple horizons (and therefore, populations separated in both time and space) and morphological variation was not discussed. High variation in morphological ontogenetic trajectories has been reported in early theropod dinosaurs, most notably *Coelophysis bauri*, *Megapnosaurus rhodesiensis*, as well as silesaurid dinosauriforms, although these studies either did not consider osteohistology^18–20^ or found it uninformative for assessing chronological age^21^. A pioneering osteohistological analysis of *M. rhodesiensis* focused on constructing a growth curve for the taxon and did not link the age and size data with external morphological variation in the same specimens^22^. Similar to early sauropodomorphs, most of these taxa (e.g., *Asilisaurus*, *Silesaurus*) are known from multiple horizons and localities. The other known stratigraphically constrained bonebed, that of *M. rhodesiensis*, consists of disarticulated specimens that provide morphological information for one or a few elements (not whole individuals) at a time^23^. Therefore, although there are strong indicators that early dinosaurs are characterized by high levels of intraspecific, population-level variation in ontogeny, the evidence for this is often incommensurable (e.g., osteohistology vs. skeletal morphology)^24^, with samples taken from fossil assemblages representing multiple populations that may be separated by vast intervals of time. This risks conflating inter-population or even interspecific differences in growth trajectories with those that may occur within a single population.

**Figure 1:**
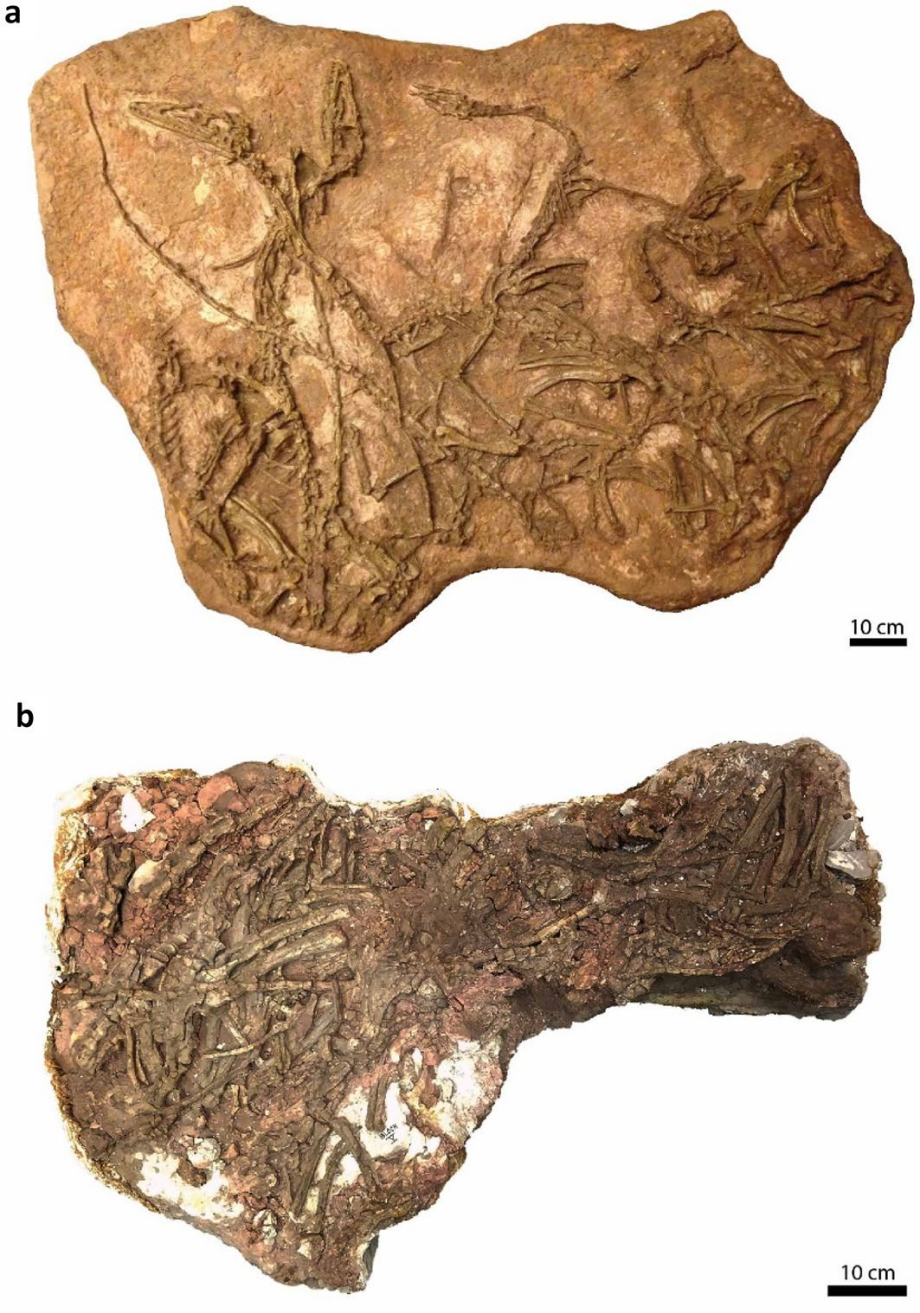
The *Coelophysis bauri* population preserved at the *Coelophysis* Quarry, Ghost Ranch, New Mexico is temporally and geographically constrained. (**a**) AMNH FARB block IX and (**b**) AMNH FARB block V^25^ from which the majority of *Coelophysis bauri* specimens were histologically sampled for this analysis. Note the articulated and overlapping nature of most of the individuals, suggesting that they died and were buried by sediment within a short span of time. [Photo credit for **b**: C. Mehling, © 2021 American Museum of Natural History. All rights reserved].

To assess whether highly plastic growth patterns were present near the origin of Theropoda and Dinosauria and to determine whether body size, gross morphology, and histological indicators of maturity correlate, we selected the bonebed assemblage of the theropod dinosaur *Coelophysis bauri* from the Upper Triassic ‘siltstone member’ of the Chinle Formation at Ghost Ranch, New Mexico (Fig. 1) as the most informative study system. Previous work identified this bonebed as a catastrophic assemblage preserving a cross-section of an approximately 200-million-year-old *Coelophysis* population^25–28^. Subsequent workers discovered unusually high amounts of variation in the growth trajectories of external morphological features, such as bony fusions and muscle scars^19,20,26,29^. Therefore, the *Coelophysis* bonebed affords a unique opportunity to conduct one of the most extensive single-element bone histology studies yet of a single population of non-avian dinosaur. We analyzed the long bone histology of 24 individuals spanning nearly the entire preserved size range to test the prediction that this population of *Coelophysis bauri* had similar levels of variation as reported for other early dinosaurs and to examine the relationship between external morphological features, body size, and age. The stratigraphic constraint, relative lack of time-averaging, articulated skeletons, and large sample size make this population of *Coelophysis* a uniquely situated study system to broadly interpret the evolution of variation and ontogeny along the avian stem.

## Results

**Figure 2:**
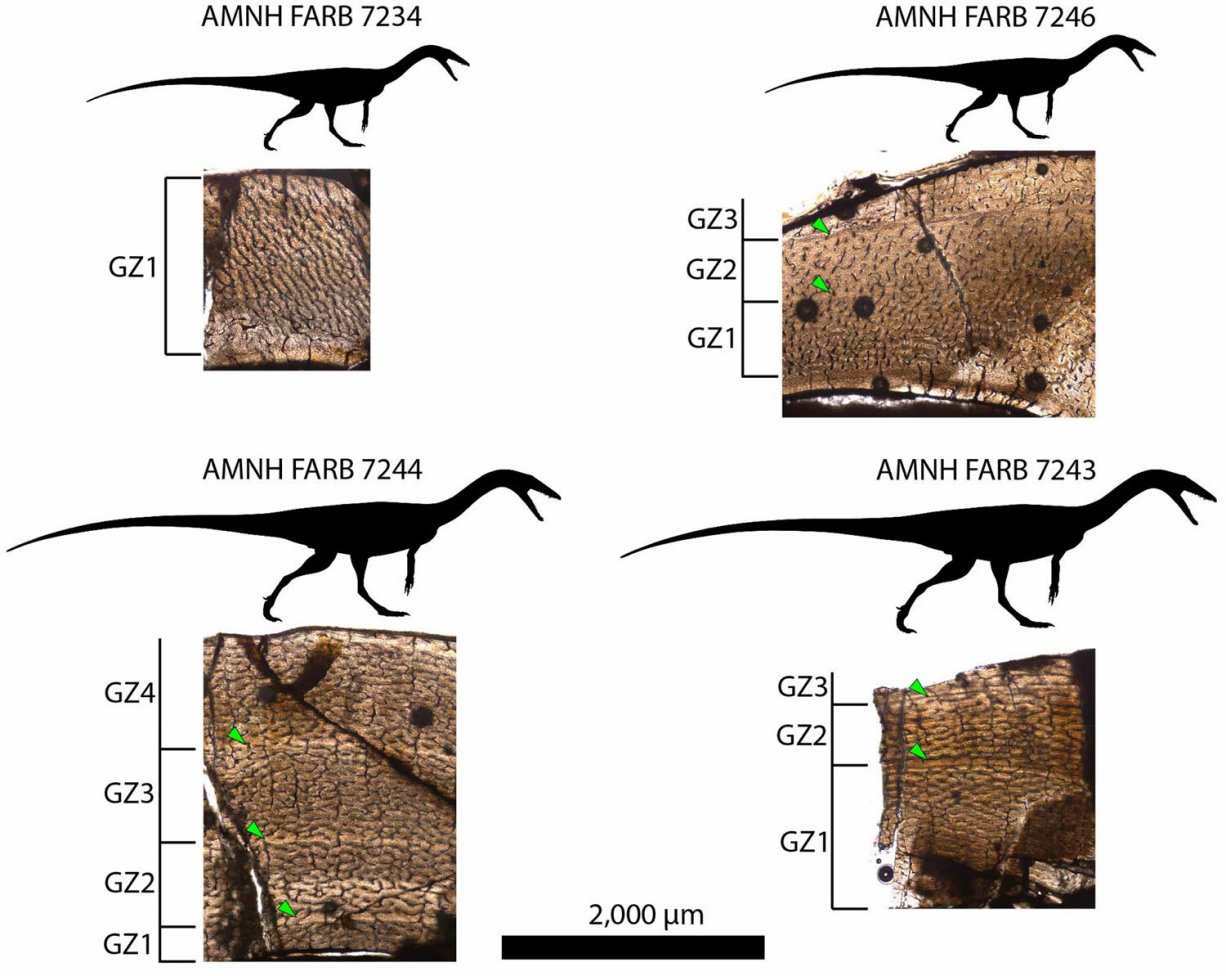
Representative *Coelophysis bauri* tibia thin sections from the *Coelophysis* Quarry at Ghost Ranch, New Mexico. All thin sections are presented at the same scale. Silhouettes were scaled to the femur length of the specimen. Green triangles point to growth marks (LAGs and annuli) that define growth zones (GZ). Silhouettes by Scott Hartman (phylopic.org, Made available through a CC BY 3.0 license https://creativecommons.org/licenses/by/3.0/).

**Figure 3:**
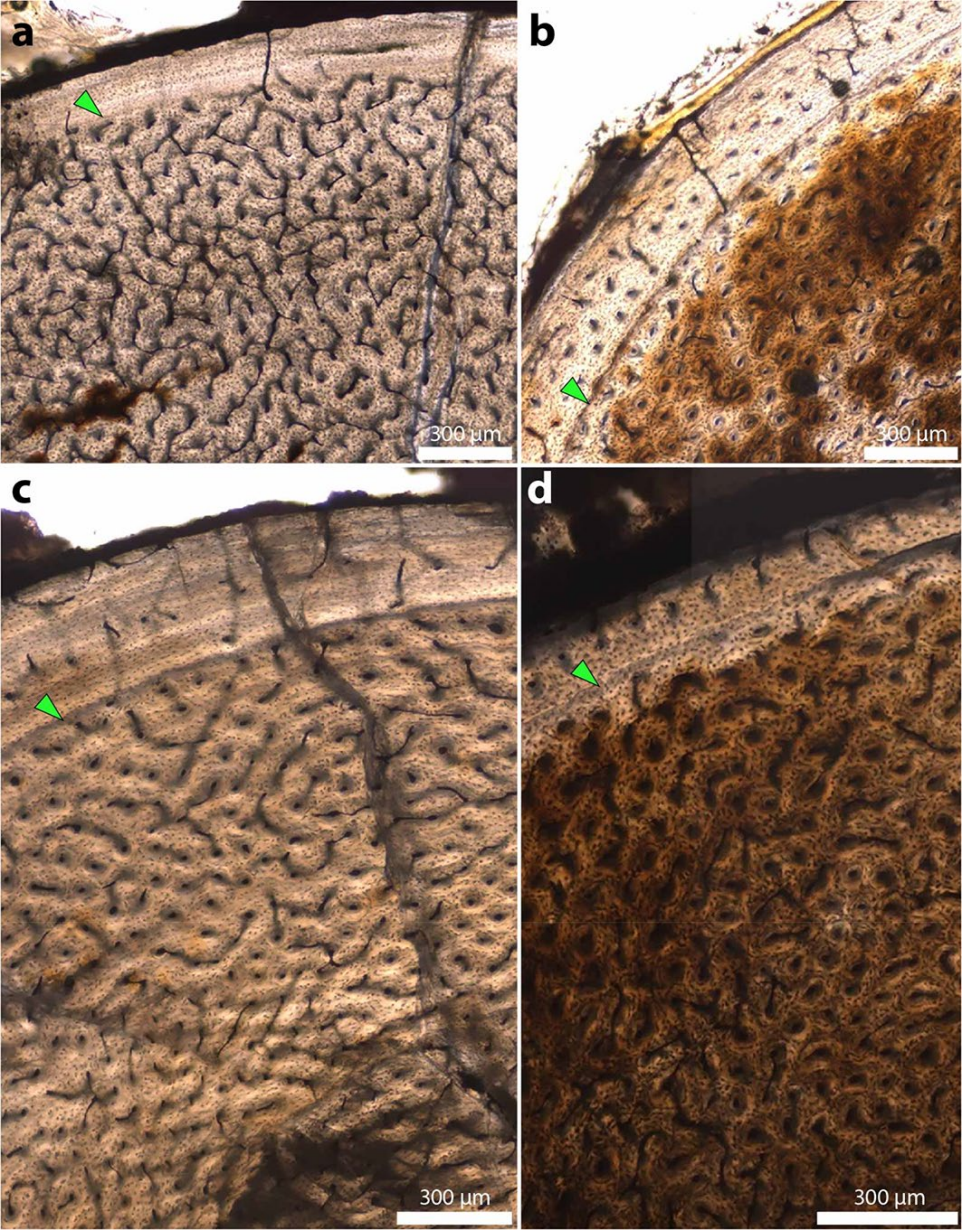
Cortices of *Coelophysis bauri* tibiae show evidence of slowed growth in individuals from different age classes. (**a**) YPM VP 41197, showing an abrupt transition from woven bone to parallel-fibered or lamellar bone in the outermost cortex. **b** AMNH FARB 7238, showing reduced vascular density after a LAG in the outermost cortex. (**c**) AMNH FARB 7251, showing an abrupt transition from woven bone to parallel-fibered or lamellar bone in the outermost cortex. A possible LAG between the marked LAG and the periosteal surface was not included in our quantitative analyses because it is highly localized to this portion of the tibia. (**d**) MCZ VPRA (Harvard Museum of Comparative Zoology Vertebrate Paleontology collection) 4332, showing reduced vascular density after a LAG in the outermost cortex, (**a**, **c**) are from four- and three-year-old individuals, respectively (based on maximum growth mark count), (**b**, **d**) are from one-year-old individuals. [Photo credit for **d**: Museum of Comparative Zoology, Harvard University, © President and Fellows of Harvard College].

Histological terminology follows^30–32^. The cortical bone of all sampled *Coelophysis bauri* tibiae consists predominantly of woven bone, with localized patches of parallel-fibered bone (Figs. 2, 3). Vascular canals are primarily longitudinal to reticular, with reticular canals more common in the mid-cortex of the larger specimens. Most tibiae, regardless of size, show a transition from reticular to longitudinal canals from the internal to external cortex. Eight tibiae, spanning a wide portion of the total circumference range, show a reduction in vascular density towards the outermost cortex (Supplementary Figs. 3, 6, 9, 13, 17–19, 21). This reduction is sometimes accompanied by an abrupt shift to parallel fibered or lamellar bone in the outermost cortex (Fig. 3a,c, Supplementary Figs. 6, 13, 21), whereas in the other tibiae there is only a gradual shift or no change in bone tissue type in this region (Fig. 3b,d). An inner circumferential layer of lamellar bone lines the medullary cavity of nearly all specimens. Some tibiae and fibulae, particularly the distally-sectioned AMNH FARB (American Museum of Natural History Fossil Amphibians, Reptiles, and Birds collection) 7247 (Supplementary Fig. 11), preserve evidence of cortical drift in the form of compacted coarse cancellous bone^33^ in the inner cortex, but this is not common overall at the midshaft position of the majority of the thin sections. Lamellar bone surrounds the vascular canals to form primary osteons in all specimens, and this is most visible in longitudinal canals. All specimens lack secondary osteons.

Growth marks are represented by both annuli, representing temporary decreases in growth, and lines of arrested growth (LAGs) representing temporary cessations of growth. There is no clear pattern to the occurrence of one type or the other. These growth marks are often localized and not continuously traceable around the entire circumference of the bone. As the histology is well-preserved overall, with little obvious diagenetic alteration or biological remodeling in most specimens, we hypothesize that this signifies differences in cortical bone deposition rate around the periosteal surface of a bone during the time interval represented by the growth mark. No tibia contains more than three growth marks (Supplementary Table 1), and none exhibit multiple closely spaced LAGs in the outermost cortex indicating skeletal maturity and near-cessation of growth, which often characterize the external fundamental system (EFS), e.g., ref.^34^. Three tibiae, YPM VP (Yale Peabody Museum Vertebrate Paleontology collection) 41197 (Fig. 3a), AMNH FARB 7251 (Fig. 3c), and AMNH FARB 7238 (Supplementary Fig. 6), have parallel-fibered or lamellar bone in their outermost cortex, similar to the outer circumferential lamellae (OCL)^32,33^. Although this poorly vascularized cortical bone lacks numerous closely spaced growth marks as in some OCLs or EFSs and it is not fully continuous along the outermost cortex, we consider it to represent an incipient shift to only incremental increases in cortical thickness, which may indicate that the three individuals had reached asymptotic growth prior to death.

Woven bone and longitudinal to reticular vascularization are common among the fibulae, and some also exhibit radial vascular canals. LAGs and annuli are also expressed in the fibulae, with a maximum of four (or possibly six) growth marks among the fibulae (Supplementary Table 1). All fibulae lack an EFS. The only sectioned femur, CMNH (Cleveland Museum of Natural History) 10971 #1, contains an EFS beginning with its fourth LAG (Supplementary Fig. 23), indicating that it had reached its growth asymptote prior to death. The femoral LAG count is consistent with the four to six growth marks in the fibula of CMNH 10971 #1 (Supplementary Fig. 24, Supplementary Table 1).

**Figure 4:**
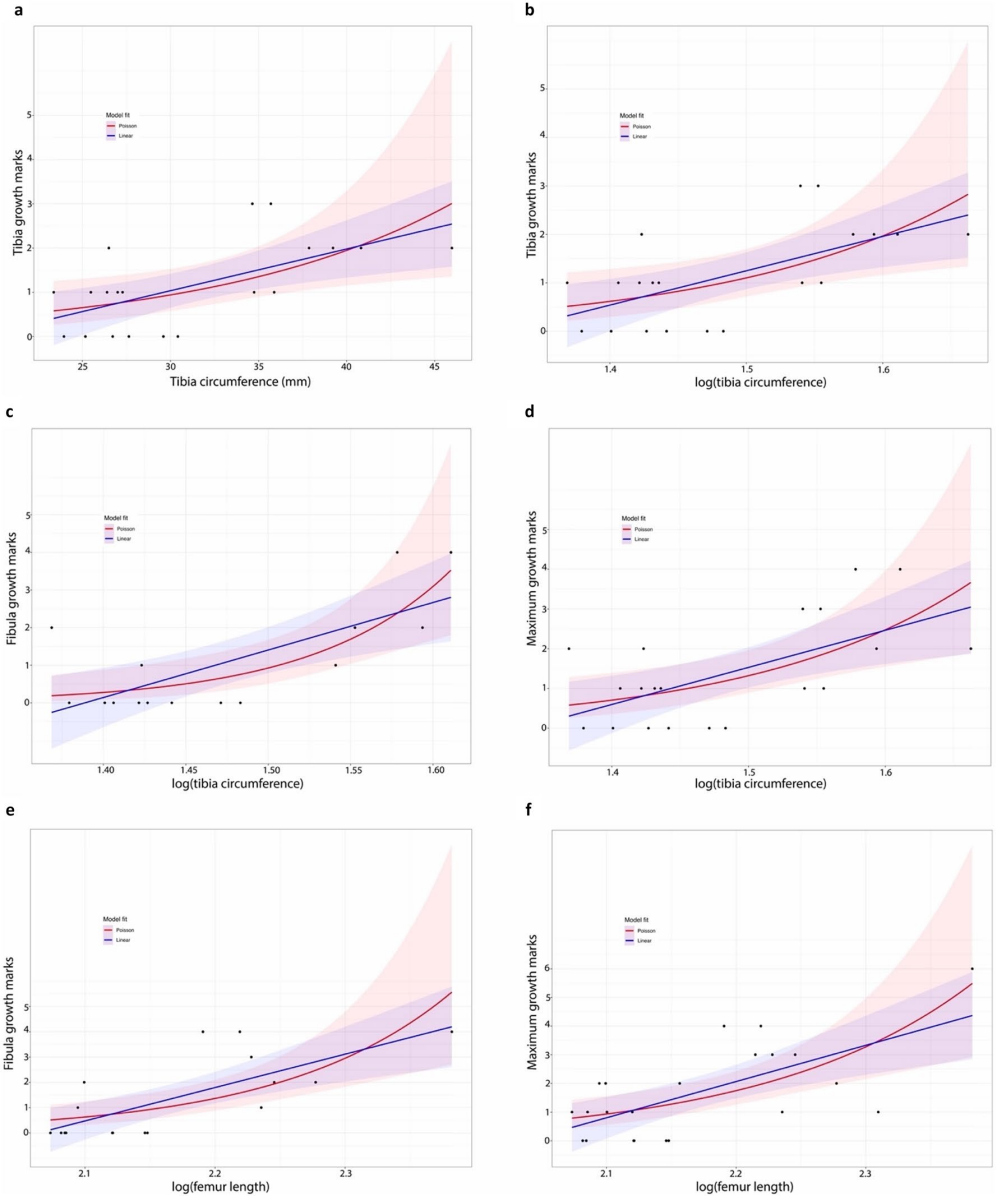
Body size proxies (tibia circumference or femur length) and the number of growth marks (an age proxy) have a significant, but highly variable, relationship in *Coelophysis bauri*. (**a**) Tibia circumference versus number of tibia growth marks. Linear: *p* = 0.0160, R^2^ = 0.2688; Poisson: *p* = 0.0304, pseudo-R^2^ = 0.1997. (**b**) Log(tibia circumference) versus number of tibia growth marks. Linear: *p* = 0.00388, R^2^ = 0.3787; Poisson: *p* = 0.0160, pseudo-R^2^ = 0.2821883. (**c**) Log(tibia circumference) versus number of fibula growth marks. Linear: *p* = 0.00261, R^2^ = 0.5145; Poisson: *p* = 0.000895, pseudo-R^2^ = 0.4777833. (**d**) Log(tibia circumference) versus maximum number of growth marks (either tibia or fibula). Linear: *p* = 0.00136, R^2^ = 0.5061; Poisson: *p* = 0.00024, pseudo-R^2^ = 0.3376748. (**e**) Log(femur length) versus number of fibula growth marks. Linear: *p* = 0.00097, R^2^ = 0.5269; Poisson: *p* = 0.000172, pseudo-R^2^ = 0.3812659. (**f**) Log(femur length) versus maximum number of growth marks (either tibia or fibula). Linear: *p* = 0.000802, R^2^ = 0.4375; Poisson: *p* = 0.000247, pseudo-R^2^ = 0.3467714. CMNH 10971 #1 has four to six growth marks in its fibula, so was plotted with four in (**e**) and six in (**f**). Blue shaded areas are the 95% confidence intervals for the linear regressions; those in red are the 95% confidence intervals for the Poisson regressions.

The age distribution is skewed towards individuals with either zero or one tibial growth mark. Those with two growth marks are the next most abundant, and those with a minimum of three are the least common (Table S1). Comparing growth mark count [a proxy for ontogenetic age, e.g., ref.^31^] against both the raw value and logarithm of tibia circumference (a proxy for body size) reveals that the two are poorly correlated (Fig. 4). This poor correlation with tibia circumference holds true no matter whether growth mark counts from the tibia (linear R^2^ = 0.38, *p* = 0.004; Poisson pseudo-R^2^ = 0.28, *p* = 0.016), fibula (linear R^2^ = 0.51, *p* = 0.0026; Poisson pseudo-R^2^ = 0.48, *p* = 0.0009), or the maximum count from either element (linear R^2^ = 0.5, *p* = 0.0014; Poisson pseudo-R^2^ = 0.34, *p* = 0.0002) are used (Fig. 4). The logarithm of estimated femur length (another proxy for body size) is similarly poorly correlated with maximum growth mark count (linear R^2^ = 0.44, *p* = 0.0008; Poisson pseudo-R^2^ = 0.35, *p* = 0.0003) (Fig. 4). Deviance goodness of fit tests for all Poisson regressions returned *p* values > 0.05, indicating the Poisson models have good fit. The fact that both the linear and Poisson regressions have significant *p* values (*p* < 0.05), but low R^2^ values, indicates that while the body size and age proxies have a general positive correlation, the percentage of variance in age explained by body size is low.

**Figure 5:**
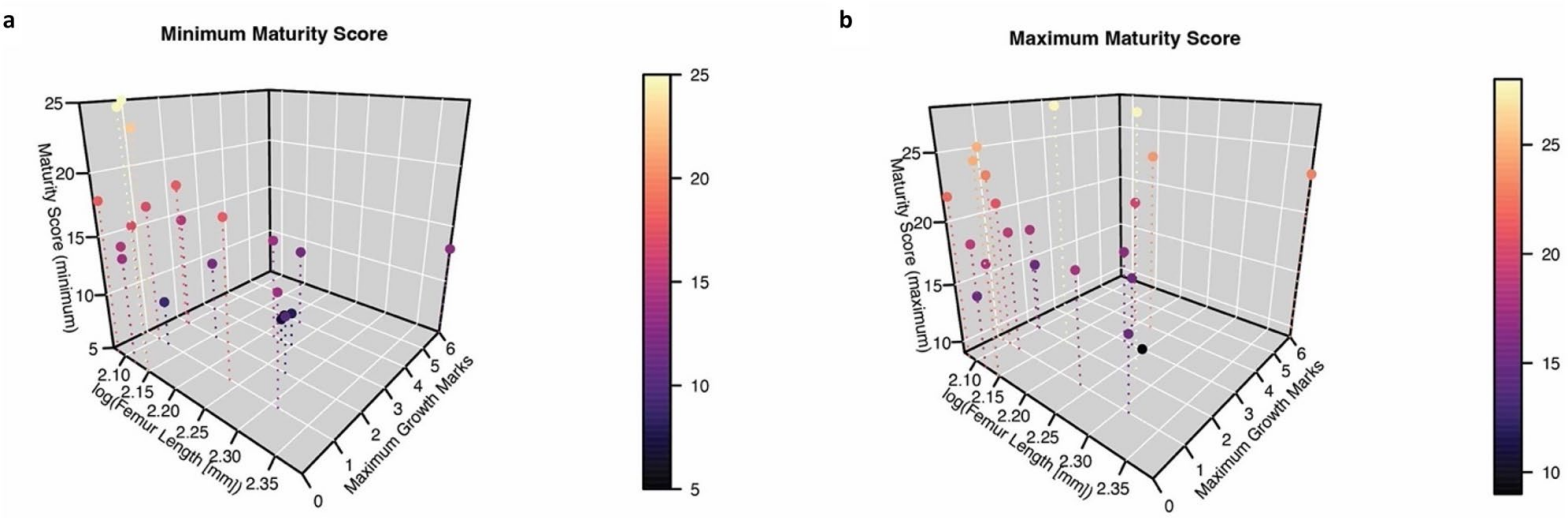
Ontogenetic variables (size, age, and external morphological maturity) in *Coelophysis bauri* are not strongly correlated with each other. (**a**) log (femur length) versus maximum growth mark count versus minimum maturity score. (**b**) log (femur length) versus maximum growth mark count versus maximum maturity score. Maturity scores were derived from muscle scar and bony fusion data by Griffin^20^. Maturity scores symbolized by color scale. All variables increase from the bottom left corner of each graph to the upper right corner. Note that the data points collectively fail to follow this trajectory, implying that increases in the three metrics of maturity are not strongly correlated through time.

Comparison of ontogenetic ages derived from growth mark counts with body size and morphological maturity scores^20^ reveals a poor correlation between all three metrics (Fig. 5). Maturity score—a measure of morphological maturity reconstructed from ontogenetic sequence analysis (OSA) of muscle scar and bony fusion characters^20^—shows no steady directional relationship with either age or size. Specimens at both the smaller and larger ends of the size range have a wide spread of maturity scores. Although some of this variation may be explained by the uncertainty (i.e., wide range) of maturity score reconstruction in some specimens, the overall pattern of variation is the same whether the minimum, maximum, or median reconstructed maturity scores are considered (Fig. 5 and Supplementary Fig. 26). This disjunct between size, age, and morphological maturity holds true for specimens with both low and high growth mark counts.

Growth zone thicknesses measured along transects of the tibia cortices (Supplementary Figs. 1–25) are highly variable from one zone to the next in *Coelophysis bauri*, similar to the condition in *Massospondylus carinatus*, but unlike the relatively constant or decreasing spacing after the first growth zones in *Alligator mississippiensis* and *Maiasaura peeblesorum*, respectively (Fig. 6).

**Figure 6:**
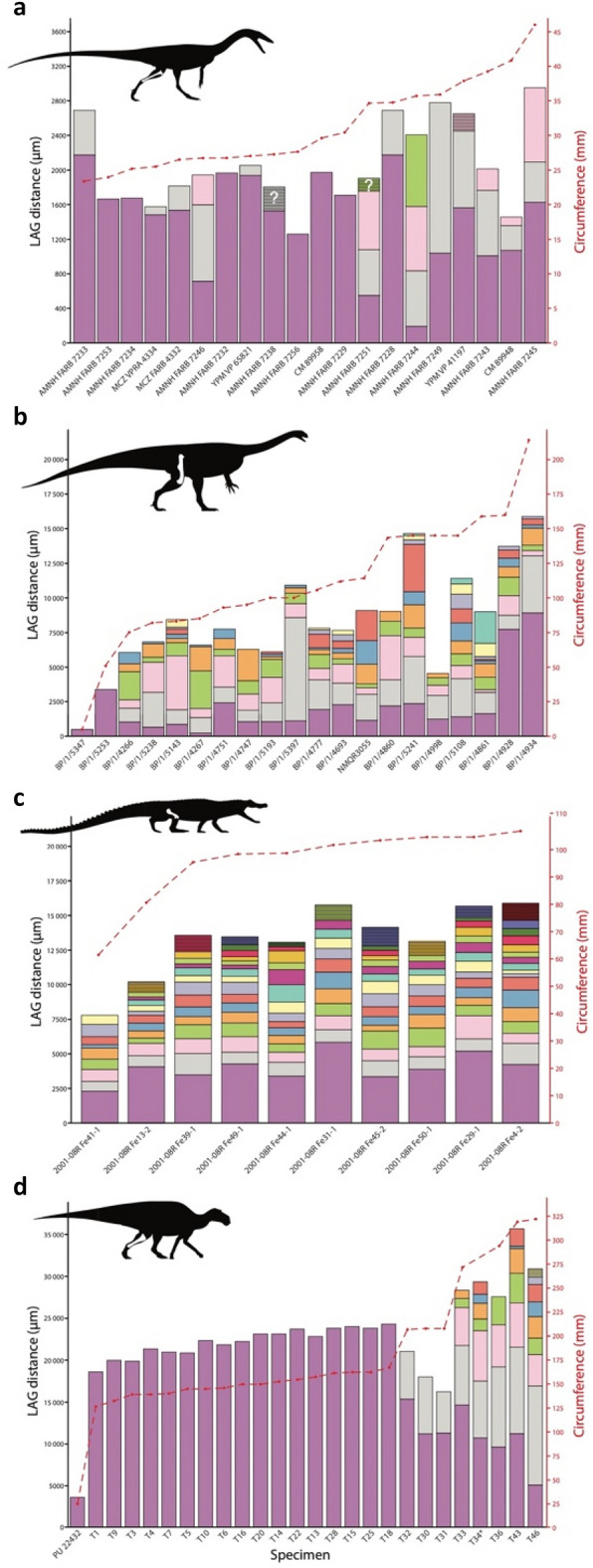
Early dinosaur growth mark spacing is more variable than that of other archosaurs. Growth mark spacing in (**a**) *Coelophysis bauri*, (**b**) *Massospondylus carinatus*, reprinted from ref.^14^ with permission of the authors, (**c**) *Alligator mississippiensis*, and (**d**) *Maiasaura peeblesorum*. Outer circumferential layers (OCLs) or external fundamental systems (EFSs) are indicated with closely spaced lines in the outermost growth zone. Skeletal drawings/silhouettes provided courtesy of Scott Hartman (www.skeletaldrawing.com), who retains ownership of these images.

## Discussion

### The *Coelophysis bauri* population consists primarily of skeletally immature individuals

The poor correlation between growth mark count and size in *Coelophysis bauri* indicates that it would be difficult to predict the ontogenetic age of a specimen given either its tibia circumference or femur length (Fig. 4). Although more speculative growth curves have been reconstructed for *C. bauri* without the use of histology^28^, our results suggest that statistically defined size classes cannot be used as a reliable proxy for age in this taxon. The amount of size variation within the population through growth and the lack of histological evidence for skeletal maturity in the majority of *C. bauri* specimens currently preclude the construction of a well-constrained growth curve^11^.

Our calculations of bone deposition rate are consistent with the assumption that the *C. bauri* individuals that lack growth marks are yearling or sub-yearling individuals^28^ (see Supplementary Text). Some specimens (Fig. 3b,d) show decreased vascular density towards the outer surface, which indicates decreased growth rate, possibly coincident with the onset of sexual maturity or environmental hardship. However, we do not observe the gradual, cyclical vascular shifts inferred to mark the onset of sexual maturity in some ornithischians^8^. The lack of an OCL or EFS in all but four of the sampled *Coelophysis bauri* reveals that most of the population had likely not yet reached skeletal maturity. This is further supported by the lack of secondary remodeling in all specimens. The only sectioned elements that preserve a possible incipient OCL or EFS are AMNH FARB 7238 (Supplementary Fig. 6) a tibia with estimated corresponding femur length of 126 mm, AMNH FARB 7251 (Fig. 3c, Supplementary Fig. 13) a tibia with estimated corresponding femur length of 164 mm, YPM 41197 (Fig. 3a, Supplementary Fig. 21) a tibia with an estimated corresponding femur length of 165.6 mm, and CMNH 1097 #1 (Supplementary Fig. 23) a large femur estimated by Griffin^20^ to be ~ 241 mm long. This shows that skeletal maturity may have been attained at a wide range of sizes in *C. bauri*. Of 70 femora measured by a previous study^28^, five are similar in length to CMNH 10971 #1 (i.e., > 233 mm in the two largest size classes), suggesting that such large and potentially skeletally mature individuals were rarely preserved in the bonebed. Such large individuals should be prioritized for future sectioning, to test for the presence of an EFS and any correlation between large size and histological maturity.

The right-skewed age distribution for *Coelophysis bauri* differs from the left-skewed distribution of another theropod population, *Albertosaurus sarcophagus*, a tyrannosaurid from the Late Cretaceous of Alberta, Canada^35,36^. It is more similar to the right-skewed distribution for *Maiasaura*^8^. A right-skewed distribution for the *Coelophysis* bonebed is consistent with a catastrophic assemblage^36^ as previously hypothesized on the basis of taphonomic evidence and the body size distribution^27,28^, but supported here for the first time with numerical ontogenetic ages of individuals, derived from osteohistology.

### Decreases in *Coelophysis bauri* bone growth may reflect variable onset of maturity or the influence of harsh environmental conditions

The transition to longitudinal canals, reduction in vascular density, and occasional shift to parallel-fibered or lamellar bone deposition are evidence for slowing growth in individuals spanning nearly the entire size range^37,38^ (Fig. 3). This provides further evidence for variation in growth trajectories, as not all specimens experienced a decrease in growth at the same age. This may reflect (1) variable onset of a growth decrease preceding sexual or skeletal maturity at different ages, or it may reflect (2) a common, environmentally-induced decrease in growth across all age classes, perhaps brought on by drought or other harsh environmental conditions inferred on the basis of sedimentological and taphonomic evidence to have preceded and/or caused the deaths of the individuals^27^. If the latter environmentally induced decrease was the case, then it provides further support for the *Coelophysis* bonebed having been generated by a single mass mortality event that affected individuals across a range of sizes^25,27^, as well as evidence of abrupt, environmentally imposed shifts in the individual life histories of most animals in the population (i.e., developmental plasticity sensu ref.^14^). These hypotheses are not necessarily exclusive and are difficult to test given the current sample. Nevertheless, it is indicative of either genetic or environmentally induced alteration of growth trajectories among the Ghost Ranch *Coelophysis* population. Our data are also consistent with the hypothesis that developmental plasticity, if present, may have proven advantageous for the survival of early neotheropod and sauropodomorph clades as they passed through unstable environmental conditions during the end-Triassic extinction to survive into the Early Jurassic^14,19^.

### Body size, osteohistological, and morphological indicators of maturity are poorly correlated in *Coelophysis bauri*

Most past work on dinosaur ontogeny examines osteohistology, body size increases, or external morphological indicators of maturity (e.g., bone processes, fusion, muscle scars, texture) in isolation. While many studies plot chronological age as determined by annual growth mark counts against measures of body size, few studies integrate all three ontogenetic variables sensu ref.^39^ to test for congruence in the relative ages they imply for a set of specimens. Notable exceptions are works which correlated histological, gross morphological, and body size maturity indicators for diplodocid sauropods and *Tyrannosaurus rex*^40,41^. Because of the potential for both body size and external morphological characters to change independently of age^6,19,20,42^, histological skeletochronology provides an essential test for the usefulness of external morphological characters as a proxy for the relative and/or absolute ages of individual specimens.

The overall poor correlation between age, body size, and external morphological features in *Coelophysis bauri* shows that, because of variability in ontogenetic trajectories, the latter two can vary a great deal for a given ontogenetic age (Fig. 5). Therefore, in *Coelophysis*, growth mark count is the most reliable means of aging specimens, as it is the only one of the three documented to consistently correlate with chronological age among a broad range of extant tetrapods^43,44^. Further studies of this kind are needed on a taxon-by-taxon basis to determine whether this disjunction is a general pattern for early dinosaurs, but previous work suggests this to be the case (see below).

Ontogenetic sequence analysis works under the assumption that all individuals in a population would eventually develop mature morphological character states given a sufficiently long lifespan^45^. However, early-diverging theropod and sauropodomorph dinosaur species typically lack osteohistologically mature representatives compared to later members of these clades. Perhaps some characters in some individuals never pass to the mature state because the individuals attained sexual maturity before mature histological and external morphological characters had an opportunity to arise during development^46^. This implies that early-diverging theropods and sauropodomorphs, in contrast to later members of these clades, adopted a life history strategy characterized by quick attainment of sexual maturity by most individuals in a population, followed by death before any potential selective advantages or disadvantages of the mature character states could be conferred. This life history strategy may call into question a fundamental assumption of ontogenetic sequence analysis; namely that all individuals in a population would eventually converge on the same suite of mature character states given a sufficiently long lifespan. Instead, reconstructed ontogenetic trajectories would end in divergent places after starting at a common origin, producing a ‘flower-shaped’ OSA network diagram, rather than the converging, oval-shaped diagrams previously reconstructed for *Coelophysis bauri*^19,20^.

### Highly variable growth trajectories were a distinct feature of early dinosaur life histories

Some of the variation in growth trajectories among *Coelophysis bauri* may be explained by sexual differences^23,26^ but this is extremely difficult to test^47–51^. Recent studies found no morphological support for the presence of sexual dimorphism in the *C. bauri* population^19,20,51^. Unless exceptional evidence of the animals’ sex, such as medullary bone or gravid females^52,53^, is discovered to allow for a direct test of the sexual dimorphism hypothesis, we consider sexual dimorphism alone to be an unlikely explanation of all the currently available data.

Finding adequate dinosaur samples for comparison of variation with *Coelophysis* is challenging. Sampling strategies employed in previous studies that were expressly designed to construct dinosaur growth curves may have obscured individual variation unless multiple specimens in each size class were targeted. Likewise, smaller subsamples of a large (n = 52) dataset of temnospondyl amphibian femora fail to generate accurate growth curves compared to the total sample^11^, strongly suggesting that reconstructing growth curves from samples with high individual variation in growth pattern is precarious. Growth curves have been reconstructed for well-sampled (n >  > 52) extant species with considerable size variation through ontogeny^54–56^, suggesting that if such high sample sizes of fossil taxa were obtained, well constrained growth curves for highly variable fossil taxa might then be reconstructed. Until such samples are available to test this, more meaningful osteohistological comparisons to the *C. bauri* data can be made with the comparably- or better-sampled dinosaurs (i.e., n ≥ 20) *Massospondylus carinatus*^14^ and *Maiasaura peeblesorum*^8^, as well as data on the extant species *Alligator mississippiensis*^8^. Comparisons of growth zone thicknesses among these four species show that *C. bauri* tibiae (Fig. 6a) and *M. carinatus* femora (Fig. 6b) have comparably high variation among individuals for their first four growth zones, even if some of the variation in the first growth zone may result from variable resorption of this zone along the medullary cavity. Adjacent growth zones often have wildly divergent thicknesses, and little pattern is discernable both across the sample and within each individual’s cortex for these taxa. This stands in contrast to the more regular pattern of steady or decreasing growth zone thicknesses through ontogeny in *A. mississippiensis* femora (Fig. 6c) and *M. peeblesorum* tibiae (Fig. 6d), respectively. While the portions of each species’ life history compared here are not exactly equivalent, restricting comparisons of variation in growth mark spacing to growth zones 1–4 for all taxa nevertheless shows that *Coelophysis* and *Massospondylus* exhibited greater variability than *Alligator* and *Maiasaura*.

These data, albeit limited, suggest that developmentally variable growth trajectories with comparable or higher variation in year-to-year growth rate than other archosaurs are ancestral for Saurischia, if not earlier nodes. *Coelophysis bauri* exhibits higher variation in year-to-year growth zone thicknesses and postcranial morphological characters than does *Alligator mississippiensis* (Fig. 6)^19^. While limited data suggest the possibility of at least some degree of developmental plasticity in phytosaurs and aetosaurs^57^, further histological sampling of extinct pseudosuchian growth series is needed to test whether the difference between *A. mississippiensis* and *C. bauri* observed here truly reflects differing ancestral growth conditions arising at the base of Pseudosuchia and Dinosauria (or even Ornithodira). The high variation seen in early neotheropods was later reduced in at least some tetanuran theropods, exemplified by *Allosaurus fragilis*, which shows a closer statistical correspondence between age and long bone circumference than *C. bauri*^58^ and less variation in postcranial morphological features throughout its growth^19^. Individual variation in growth zone thicknesses has been reported in coelurosaurs (*Tyrannosaurus rex* and ornithomimids, respectively)^59,60^; however, growth zones generally show constant or consistently decreasing thickness throughout growth in individual allosauroid and coelurosaur specimens^61^, differing from the more variable zonal thicknesses in *C. bauri* individuals*.* Greater sample sizes of age versus size data are still needed to make more meaningful comparisons between other theropods and the *C. bauri* and *A. fragilis* data.

Data on the degree of growth variation along the transition from early-diverging sauropodomorph outgroups to Sauropoda were more limited until recently, owing to the lack of extensive histological sampling of sauropod populations comparable to that undertaken for *Plateosaurus* and *Massospondylus* and the lack of LAGs in many sauropods^62^. Recently, a high degree of growth variation was found in a large sample of the sauropodiform *Mussaurus patagonicus*^63^. Some degree of variation is also known from the putative early sauropod or close sauropod outgroup taxon *Antetonitrus ingenipes*^64,65^, though the bonebed from which it was sampled also contains a second taxon^66^, potentially a confounding source of variation. Some degree of growth variation in early ornithischians is indicated by the osteohistology of five individuals of the early-diverging non-iguanodontian ornithischian *Jeholosaurus shangyuanensis*^67^, whereas size and age correlate well in a large sample of the Early Jurassic ornithischian *Lesothosaurus diagnosticus*, suggesting little developmental plasticity^68^. However, without broader sampling of Ornithischia, particularly of Triassic or Early Jurassic representatives, it remains to be seen if the comparatively low degree of variation in annual growth among *Maiasaura peeblesorum* specimens (Fig. 6) is a derived or plesiomorphic condition relative to other ornithischians.

Comparing levels of variation among early-diverging theropods and sauropodomorphs and extant birds requires special consideration, owing to distinct avian life history strategies. Whereas some palaeognath birds retain growth marks indicative of multi-year maturation^69^ or decreases in growth within the span of a year^70^, most neognath birds grow to nearly their final adult size within a year and tend to lack growth marks that define distinct growth zones^15^. Any fluctuations in the pre-asymptotic growth patterns of neognaths therefore occur within a shorter window of time and may not be as easily recognized histologically as those of non-avian dinosaurs with multi-year maturation. Some birds temporarily alter their development in response to resource limitation (induced response), whereas others respond passively (imposed response)^3,15^. The latter might be expected to show greater variation in final adult size within a population than in birds that can delay their maturation as their growth rate slows^3^. These observations may not be directly comparable to other archosaur datasets due to the narrower geographic and temporal scales over which plasticity was addressed. However, in a comparative study utilizing extant avian samples with a geographic and temporal resolution similar to that available for extant *Alligator* and fossil archosaurs, the birds exhibited less variable maturation pathways of their external osteological characters compared to other archosaurs^19^. Also, two extant bird species, *Passerculus sandwichensis*^71^ and *Diglossa carbonaria*^72^ measured from large (n > 100) samples across continent-scale geographic ranges showed comparatively narrower ranges of variation in linear size measurements than we report for the femur lengths of the single *Coelophysis bauri* population, supporting the idea that early dinosaur populations were more variable than extant bird populations in this regard.

Bird hatchlings are essentially ectothermic and shielded from many changes in their thermal environment early in their growth trajectory by parents that can provide body heat and shade that buffers hatchlings from environmental fluctuations in temperature, until the juvenile birds develop sufficient muscle mass to generate heat and insulation to maintain homeothermy^73^. Therefore, bird development, even at its earliest stages, should be relatively more shielded from the kinds of thermal environmental impacts on growth that non-avian reptiles are subject to throughout their lives. Other environmental conditions, such as food availability, may affect adults as well as their offspring. Biological factors such as a genetic component of variation, the level of parental care investment, and position within a nestling or social hierarchy may also effect changes in growth trajectories^74–76^. The longer growth duration of early saurischians compared to most extant birds means there are more opportunities for non-avian dinosaur growth to be affected by environmental or biological conditions before adult size is reached, providing a possible explanation for the relatively high size variance within the *Coelophysis bauri* population^15^. In turtles, lizards and some birds, environmental changes affecting the growth trajectory during the period of highest growth produce variance in body sizes that are then sustained after adulthood is reached^12,77,78^. Therefore, we can predict that older age classes of *C. bauri* than those currently sampled would also show high levels of body size variance. We further predict that this would result in increasing variance (divergence of growth trajectories of individuals) with increasing age in longer-lived taxa. However, further sampling of non-avian dinosaur and extant taxa is necessary to fully test this prediction.

Histological study of the Ghost Ranch *Coelophysis bauri* population provides an unprecedented look into the growth patterns, population structure, and individual variation within a single taxon of early dinosaur. Ontogenetic ages derived from bone histology confirm the right-skewed age distribution of the population, supporting previous interpretations that the assemblage resulted from a mass mortality event. Poor correlations between body size, age, and external morphological development indicate a high degree of intraspecific variation in growth trajectories, which is uniquely elevated in early saurischians compared to later dinosaurs and some non-dinosaurian archosaurs.

## Methods

### Histological analysis

We removed midshaft segments approximately 10–30 mm in proximodistal length from the tibiae of 22 *Coelophysis bauri* individuals from the Ghost Ranch bonebed from the Upper Triassic Chinle Formation (Fig. 1 and Supplementary Figs. 1–25). See Supplementary Figs. 1–25 for more precise sampling locations. One specimen, AMNH FARB 7247, was sampled distally, closer to the ankle, and was excluded from our further quantitative analyses because much of its original microstructure was obliterated by cortical drift and deposition of compacted coarse cancellous bone (Supplementary Fig. 11). Another specimen, ROM (Royal Ontario Museum) 72668, was also excluded from our quantitative analyses because of an unusual bony callus that may have formed in response to abnormal loading and/or pathology. The etiology of this bony callus is the subject of ongoing study. Sixteen fibulae were sectioned along with their adhering tibiae, with the fibula of AMNH FARB 7247 likewise excluded from further quantitative analysis because of the effects of cortical drift. Two additional individuals, CMNH 10971 #1 and CMNH 10971 #5, are represented by a sectioned femur and fibula, and a sectioned fibula, respectively. The specimens span the entire size range (based on measured or estimated femur length) of individuals in the bonebed^28^. Following removal, we molded and cast these specimens to preserve a record of their morphological details. We embedded the specimens in Epo-Tek 301-2 optically transparent epoxy, sectioned into 1.2–1.5 mm thick wafers with a Buehler Isomet 1000 precision saw. The wafers were attached to pre-frosted standard (27 × 46 mm) glass petrographic slides. We then ground them to optical transparency using Extec 600 and 800 grit paper. Specimens were immersed in oil before viewing and photography under both a Leitz Laborlux 11 Pol S petrographic microscope and a Zeiss Axio Imager 72 Automated Microscope Imaging System (to capture the entire cross section in a series of stitched images). For details on modified methods used to prepare the CMNH and ROM specimens, see the Supplementary Notes. We used cross-polarized light both with and without a lambda plate to examine differences in birefringence among tissue types and growth marks that aided in their identification. We measured all thin section photomicrographs using ImageJ and FIJI image analysis software^79,80^.

We defined growth marks as lines of arrested growth (LAGs) (a dark line representing a depositional hiatus) or annuli (narrow, avascular bands of parallel-fibered bone marking a decrease in growth). Tibia circumferences and growth zone thicknesses were measured from the stitched images using ImageJ and FIJI. In most cases, the entire circumference of the bones were preserved, only fractured into fragments that were displaced by crushing of the shaft. Therefore, we traced the length of the outer, periosteal surface of each fragment using FIJI, then added the lengths of all the fragments to reconstruct the total circumference. In cases where fragments were obviously missing, we visually estimated how much of a gap would have remained and measured across this between the two preserved points. The curvatures of the endosteal surfaces and growth marks of the fragments also aided us in determining whether fragments were missing. We acknowledge that this method is imprecise, but in most cases the gaps involved are small and unlikely to have greatly affected our conclusions. For each specimen, tibia circumference was graphed against growth mark counts (a proxy for age in years) from (i) the tibia, (ii) the fibula (if preserved), and (iii) the maximum number of growth marks preserved by either the tibia or the fibula.

Retrocalculation to estimate the number of growth marks that may be missing due to medullary cavity expansion was not attempted, as the *C. bauri* specimens are poorly suited for any of the various methods. Section stacking^81^ was not advisable, as the sample contained no hatchling or skeletally mature specimens to constrain estimates of missing growth marks. Additionally, section stacking accounts poorly for individual variation in growth^82^, something that is prominent in the *C. bauri* sample. Interval-based retrocalculation methods^83,84^ were deemed inadvisable due to the lack of constraint on medullary cavity size in the youngest (i.e., hatchling) growth stages of *Coelophysis*. While hatchling size may be negligible in comparison to adult size for larger dinosaurs, and the medullary cavity size negligible for those with narrow medullary cavities, these are not good assumptions for small theropods with expansive medullary cavities, which may have been wide even at embryonic stages^85^. Finally, the use of curve-fitting graphical retrocalculation^8,86,87^ was not possible because of the lack of skeletally mature specimens and the low number of growth marks (maximum of three), available to constrain growth for any individual. Additionally, the taphonomic crushing of specimens (sometimes with loss of portions of the cortex) and the tendency for growth marks to not be constantly visible around the entire cortex in some specimens complicate all retrocalculation methods. Therefore, we present only raw growth mark counts as the least assumption-laden alternative for aging the specimens. Specimens with no growth marks were likely in their first year of life, as it is not likely based on a priori knowledge of extant taxa that either no growth marks formed during the first year, or that all growth marks had completely eroded by the time the current cortex was deposited^43,88^. These zero-growth-mark specimens are also of a comparable size percentage relative to the total known sample as other small sub-yearling theropod individuals^89^. See Supplementary Notes for additional rationale for the yearling or sub-yearling status of these specimens.

Previous studies suggest the fibula may be a better element for aging theropods because it often preserves a more complete record of growth marks^60,82,90^. In cases where the number of growth marks in the fibula exceeded the number in the tibia, the number of fibular growth marks was used as the age proxy for our regressions of maximum growth mark count versus tibia circumference. This was true for three *C. bauri* specimens, whereas four showed a higher growth mark count in the tibia, and eight showed no difference (Supplementary Table 1). However, in many specimens the growth marks were obscured or truncated by cortical drift or the irregular shape of the fibula. We therefore concur with Cullen et al.^60^ that a multi-element approach such as ours is desirable as a check on the number of growth marks preserved in any one element.

Femur lengths for 21 of the specimens were measured or estimated using previously published linear regressions that estimate femur length from the length of other elements^20^. Another measured femur length, for AMNH FARB 7238, was obtained from Colbert^25^.

We attempted to measure growth mark spacing following the method of Chapelle et al.^14^. However, it was impossible to measure along the widest part of the tibia cortex for all specimens, owing to the discontinuous preservation of growth marks on many sections and because of crushing and fragments missing from the tibiae along fracture surfaces. Therefore, we measured along transects through the thickest portion of cortex that contained all the traceable growth marks (Supplementary Fig. 1–25). We recognize that measuring growth mark spacing at homologous locations or measuring cortical area instead of spacing is the best way to minimize variation^91^. Our approach was designed to incorporate all growth marks in each section as potentially useful age indicators. The degree to which discontinuous growth mark count and continuously traceable growth mark count correspond can be tested by future discovery of specimens in which all growth marks can be traced around the entire tibia circumference.

We obtained comparative growth mark spacing data (Fig. 6) from photomicrographs of previously prepared captive male *Alligator mississippiensis* femora thin sections (n = 10) and *Maisaura peeblesorum* tibia thin sections (n = 26)^8^. The original slides for both taxa are reposited at Museum of the Rockies, Bozeman, Montana (MOR).

### Morphological maturity scores

Morphological maturity scores were derived from the ontogenetic sequence analysis (OSA) of Griffin^20^ by compiling the range of possible semaphoronts reconstructed by the combination of postcranial morphological characters from the pelvis, sacrum, femur, tibia, tarsus, and pes from 21 of the studied *C. bauri* specimens. We used the minimum, maximum, and median maturity scores for all specimens to construct three plots of maturity score, growth mark count, and log(femur length) (Fig. 5 and Supplementary Fig. 26).

### Statistical analysis

Data points were plotted and regressions performed using R statistics software. Linear models were constructed with the lm() command; Poisson regressions were constructed with the glm() command. Plots were constructed with the package ggplot2^92^. In addition to the standard linear regression frequently used in paleohistological studies, we also fit a Poisson regression to our data (Fig. 4). Poisson regressions model count data, such as growth mark count, which has been treated as continuous data in most previous paleohistological studies because of its use as an age proxy. We present both regression models to test whether the treatment of growth mark count data affects our results. Pseudo-R^2^ for Poisson regressions was calculated by subtracting the residual deviance divided by the null deviance from 1 (McFadden’s pseudo R^2^; 1–(ResidualDeviance/NullDeviance)^93^. Deviance in a Poisson regression measures how closely our model’s predictions are to the observed outcomes. The deviance goodness of fit test returns a *p* value. This tests whether there is a statistical difference between the deviance in our observed results versus the deviance we would expect in the observed outcomes around their predicted means, under the Poisson assumption. A high *p* value (*p* > 0.05) indicates that the data and the expected data under the model are statistically indistinguishable, that there is no evidence for poor model fit.

## Supplementary Information

Supplementary Information 1.

Supplementary Information 2.

Supplementary Information 3.

Supplementary Information 4.

Supplementary Information 5.

Supplementary Information 6.

## Data Availability

All data needed to evaluate the conclusions in the paper are present in the paper and/or the Supplementary materials.
