# Supplementary material for: Osteohistology of a Triassic dinosaur population reveals highly variable growth trajectories typified early dinosaur ontogeny
Source: Sci Rep. 2022 Oct 15;12:17321. doi: 10.1038/s41598-022-22216-x (PMC9569331; doi:10.1038/s41598-022-22216-x)
Supplement: Supplementary file 1 — Supplementary Information 1. [file 41598_2022_22216_MOESM1_ESM.pdf]

SUPPLEMENTARY INFORMATION

Osteohistology of a Triassic dinosaur population reveals highly variable growth trajectories typified early dinosaur ontogeny

Daniel E. Barta*, Christopher T. Griffin, Mark A. Norell

*Corresponding author. Email: daniel.barta@okstate.edu

This Supplementary Information file includes:
Supplementary Notes
Supplementary Figs. 1-26
Supplementary Table 1
Legends for Supplementary Tables 2-4
Legends for Supplementary Data 1 and 2
Supplementary References

Other Supplementary Information for this manuscript includes:
Supplementary Tables 2-4
Supplementary Data 1 and 2

Supplementary Notes

Additional high-resolution images of supplementary figures, R code, and .csv file are available from Figshare (https://doi.org/10.6084/m9.figshare.c.6173626).

Additional Histology Methods

Cleveland Museum of Natural History (CMNH) specimens: Because sampling of a full cross-section of long bones was not possible for CMNH *Coelophysis bauri* specimens, we instead sampled fragments taken from the midshaft of long bones. For the fibula of CMNH 10971 #1, we sampled a posterior portion of the left fibula, and additionally sampled fragments from both the medial and lateral sides of the cortex of the right femur from this individual. We also sampled a fragment from the anterior half of the right fibula of CMNH 10971 #5.

We embedded the fragments in Castolite-AC resin, placing the wet resin under vacuum for ~2 minutes to ensure the resin fully embedded the samples. After the resin set, we used a Buehler Isomet 1000 diamond-bladed saw to cut 1.5-mm thick transverse wafers of the embedded bone. Following this, we polished one side of each wafer using a Buehler grinding/polishing wheel and glued the polished side to a plexiglass microscope slide using cyanoacrylate glue. We ground the glued wafer down to an acceptable thickness to see histological detail using the grinding/polishing wheel and polished when this thickness was reached. We viewed slides with an Olympus BX51 petrographic microscope under plane-polarized light, cross-polarized light, and cross-polarized light under a 530 nm gypsum wave plate. Histological images were captured with a Luminera Infinity 1 microscope camera, and whole-slide images were stitched together using Adobe Photoshop version 21.2.1.

Royal Ontario Museum (ROM) specimen: We embedded the broken distal end of ROM 72668 in Castolite AP resin and cut a wafer using a Buehler Isomet 1000 diamond-bladed saw. We frosted and glued together both the wafer and a plexiglass slide with cyanoacrylate glue. The wafer was then trimmed further with the Buehler Isomet 1000 saw. We ground the resulting thick section to an acceptable thickness to observe histological detail using a Hilquist 1010 thin sectioning machine. We polished the thin section by hand using progressively finer (600 to 1000) silicon carbide grits and water on a plexiglass plate. We viewed the specimen under plane-polarized and cross-polarized light and captured digital photomicrographs using a Nikon Az100 petrographic microscope. The photomicrographs were stitched together using Nikon Elements imaging software.

Growth Rate Calculation

To determine if *Coelophysis bauri* tibiae lacking growth marks could have grown to their preserved circumference within a year, we constructed a simple geometric model to test the assumption that they represent yearling or sub-yearling individuals. The preserved circumferences of the sampled *C. bauri* tibiae lacking growth marks are all under 31 mm (excluding the histologically abnormal ROM 72668) (Supplementary Table 1). Modeling a circular tibial midshaft with a 31 mm circumference (radius ~ 4.9 mm) gives a growth rate of 12.8 μm/day, assuming that the entire radius was deposited within a 381-day Triassic year[1]. Accounting for a possible 90-day growth hiatus [e.g., ref.[2]] gives a growth rate of 16.8 μm/day. Assuming a one-half year growth hiatus of 190.5 days gives a growth rate of 25.7 μm/day. Of

course, calculating the radius in this way does not factor in the size of the medullary cavity, so all estimates provided here are certainly overestimates of growth rate.

Alternatively, we can use the measured first growth zones to model the growth rate, though these may preserve less than the amount of bone deposited in a year, due to medullary cavity expansion. None of the measured first growth zones exceed approximately 2,300 μm (Fig. 6). Using this number for ease of calculation gives hypothetical growth rates of 6.0 (full year), 7.9 (90-day hiatus), and 12.1 μm/day (190.5-day hiatus).

All calculated rates lie within the range of fibrolamellar and lamellar/parallel-fibered bone in extant mallard ducks (*Anas platyrhynchos*)[3], and although the high growth rates of extant birds make them difficult to directly compare with *Coelophysis* growth rates they provide an upper bound of what is reasonable for given tissue types. Previous estimates of the growth rate of another coelophysid specimen [University of California Museum of Paleontology (UCMP) 129618] found a range of 15-17.5[4] or 5-20 μm/day[5], very similar to the rates we calculated. The rates are similar to the maximum calculated for the later ontogeny of tibiae of the large theropod *Tyrannosaurus rex*[6] and slower than those of the first three years of tibial growth of the large ornithopod *Maiasaura peeblesorum*[2]. This is expected for a smaller dinosaur that grew more slowly early in life[5]. Therefore, the *Coelophysis* appositional growth rates presented here do not seem unrealistically high for a dinosaur, so it is plausible that individuals with zero growth marks died sometime during their first year of life. This supports our assumption that a substantial number of growth marks are not missing due to medullary cavity expansion or other processes in the zero-growth mark specimens.

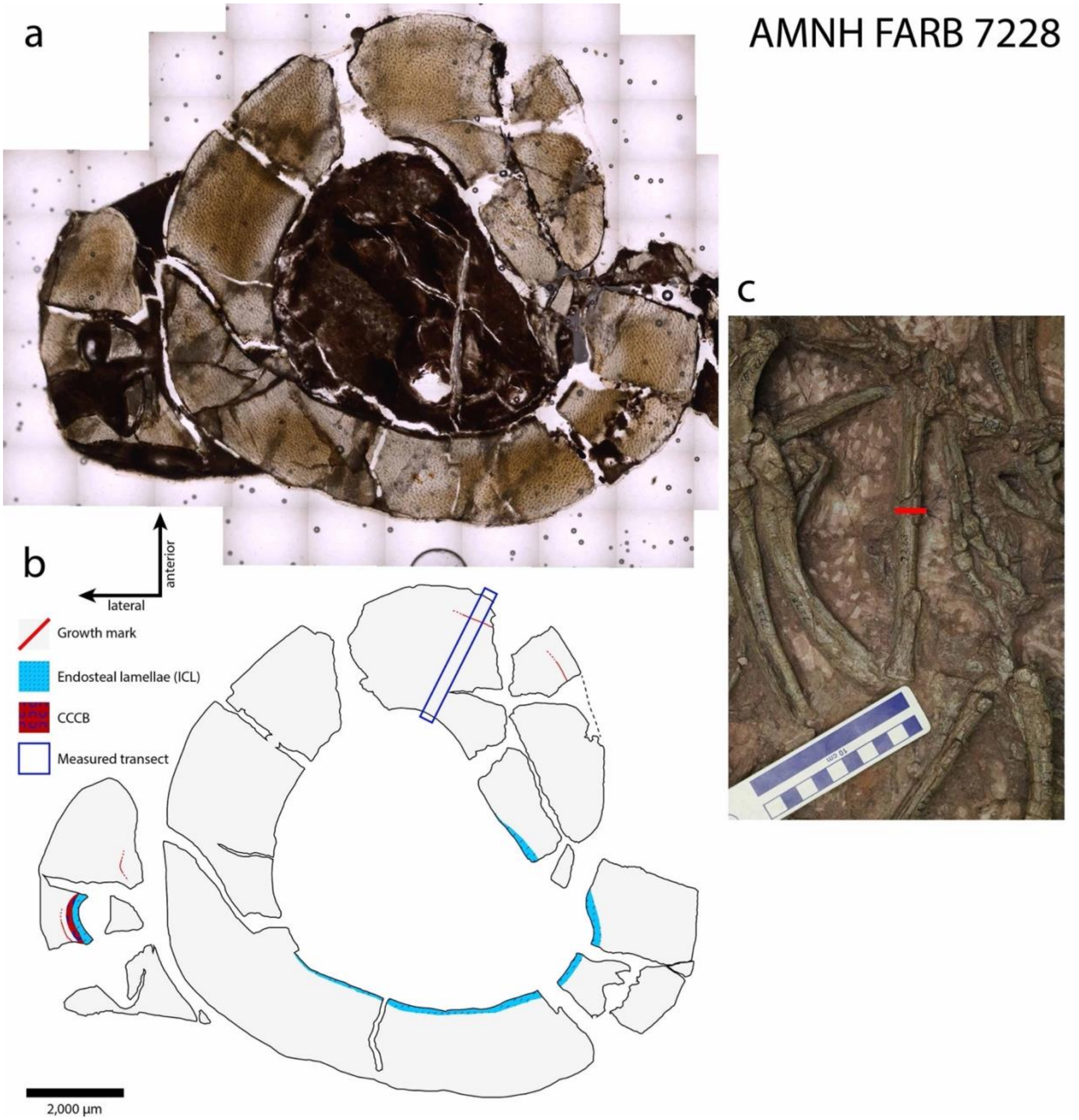

Supplementary Fig. 1 *Coelophysis bauri* left tibia and fibula AMNH FARB 7228. **a** Thin section photomicrograph. **b** Interpretive line drawing of **a**, indicating histological features and measured transect. **c** Sampling location for thin section indicated by red line. Scale bar for **a** and **b** is 2,000 µm. Each square on scale bar in **c** is 1 cm long.

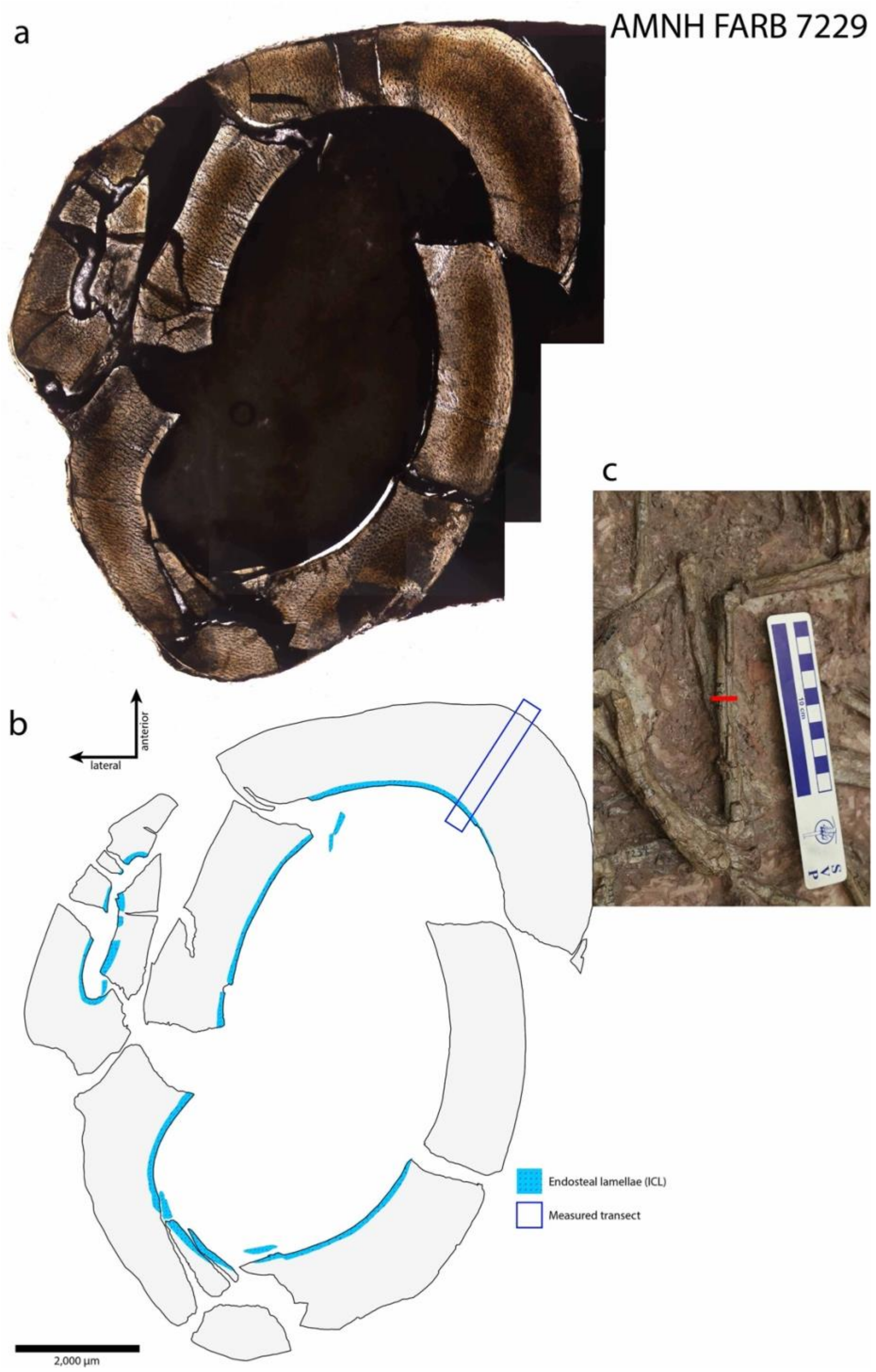

Supplementary Fig. 2 *Coelophysis bauri* left tibia and fibula AMNH FARB 7229. **a** Thin section photomicrograph. **b** Interpretive line drawing of **a**, indicating histological features and measured transect. **c** Sampling location for thin section indicated by red line. Scale bar for **a** and **b** is 2,000 µm. Each square on scale bar in **c** is 1 cm long.

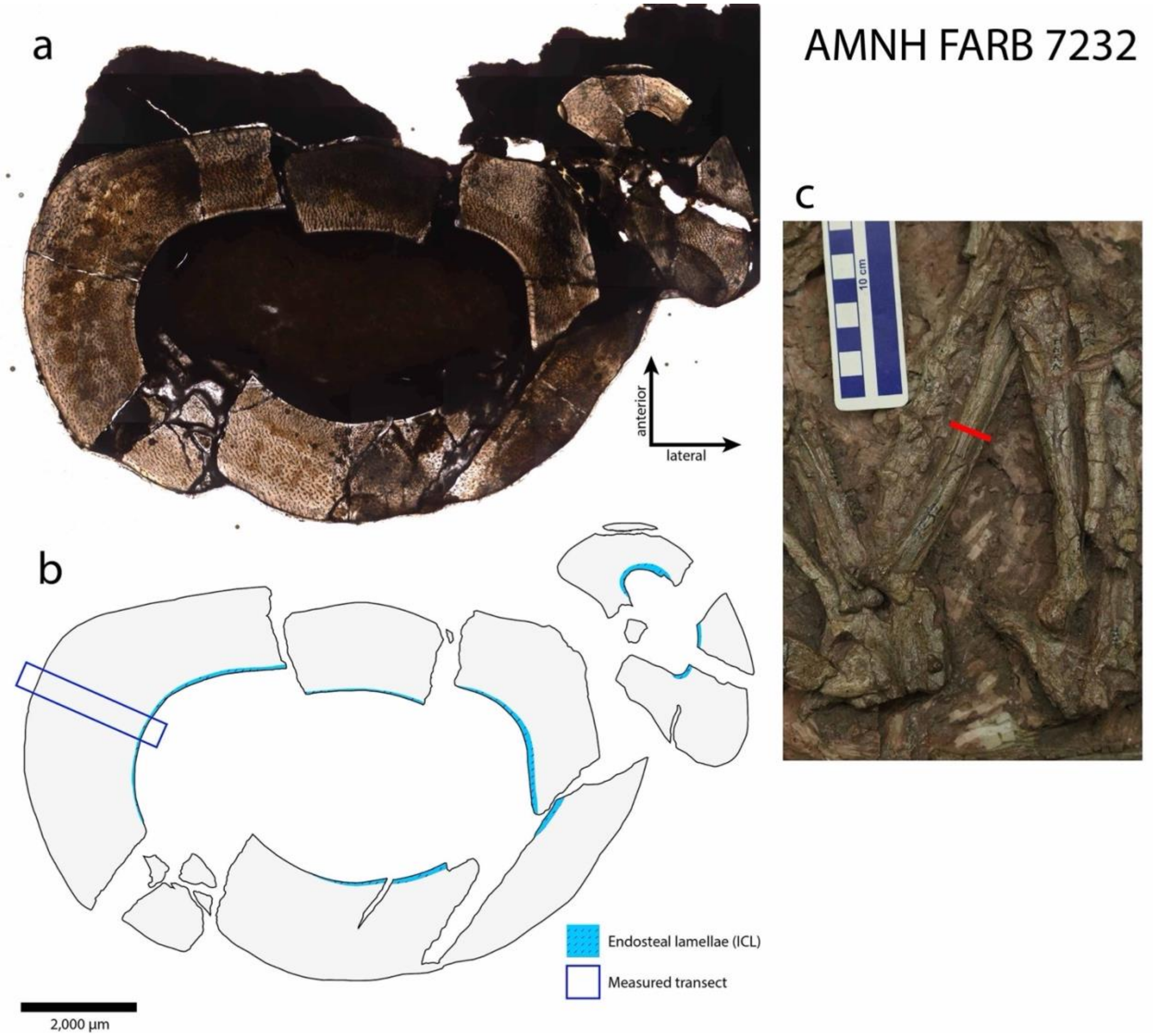

Supplementary Fig. 3 *Coelophysis bauri* left tibia and fibula AMNH FARB 7232. **a** Thin section photomicrograph. **b** Interpretive line drawing of **a**, indicating histological features and measured transect. **c** Sampling location for thin section indicated by red line. Scale bar for **a** and **b** is 2,000 µm. Each square on scale bar in **c** is 1 cm long.

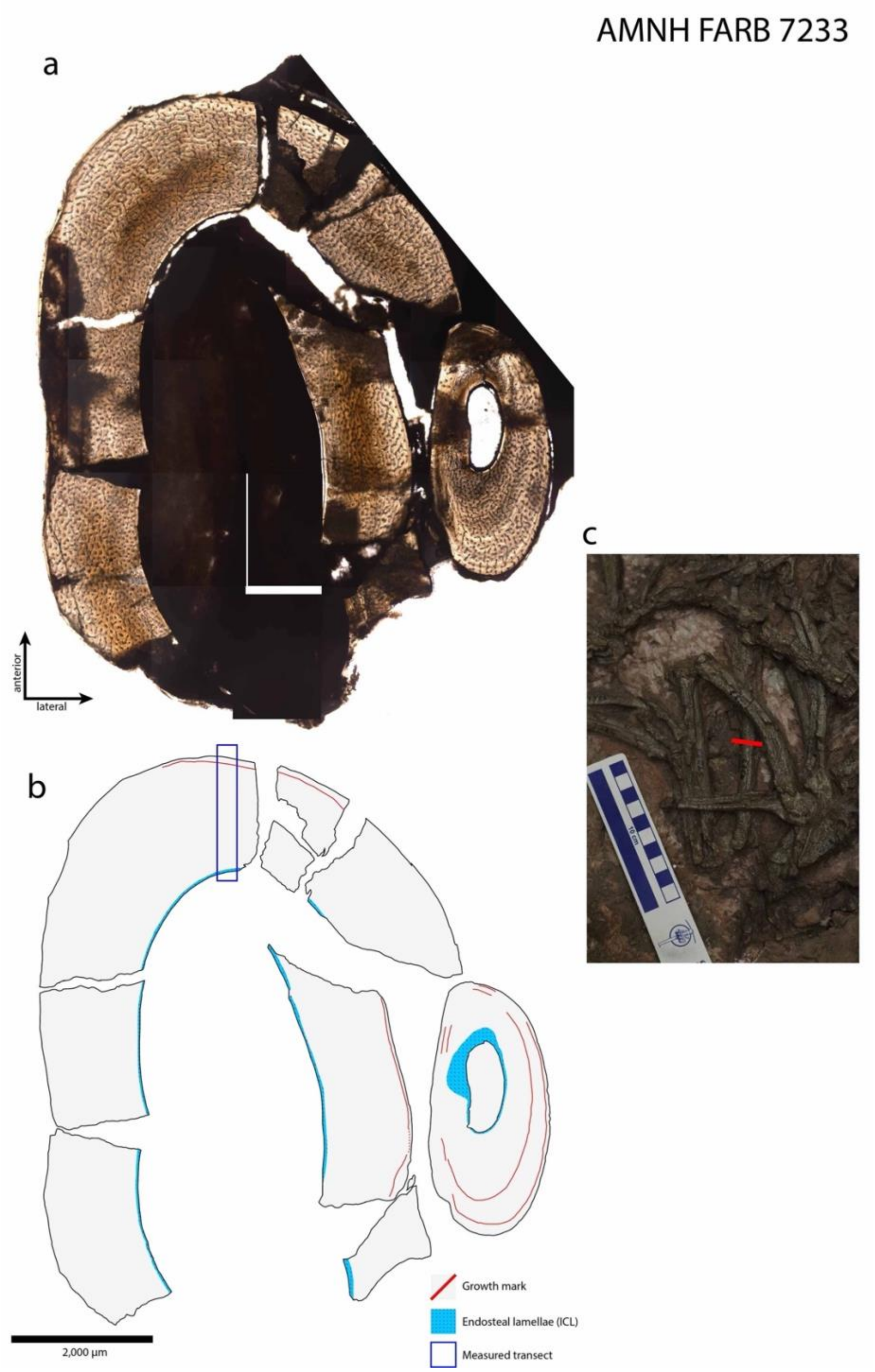

Supplementary Fig. 4 *Coelophysis bauri* right tibia and fibula AMNH FARB 7233. **a** Thin section photomicrograph. **b** Interpretive line drawing of **a**, indicating histological features and measured transect. **c** Sampling location for thin section indicated by red line. Scale bar for **a** and **b** is 2,000 µm. Each square on scale bar in **c** is 1 cm long.

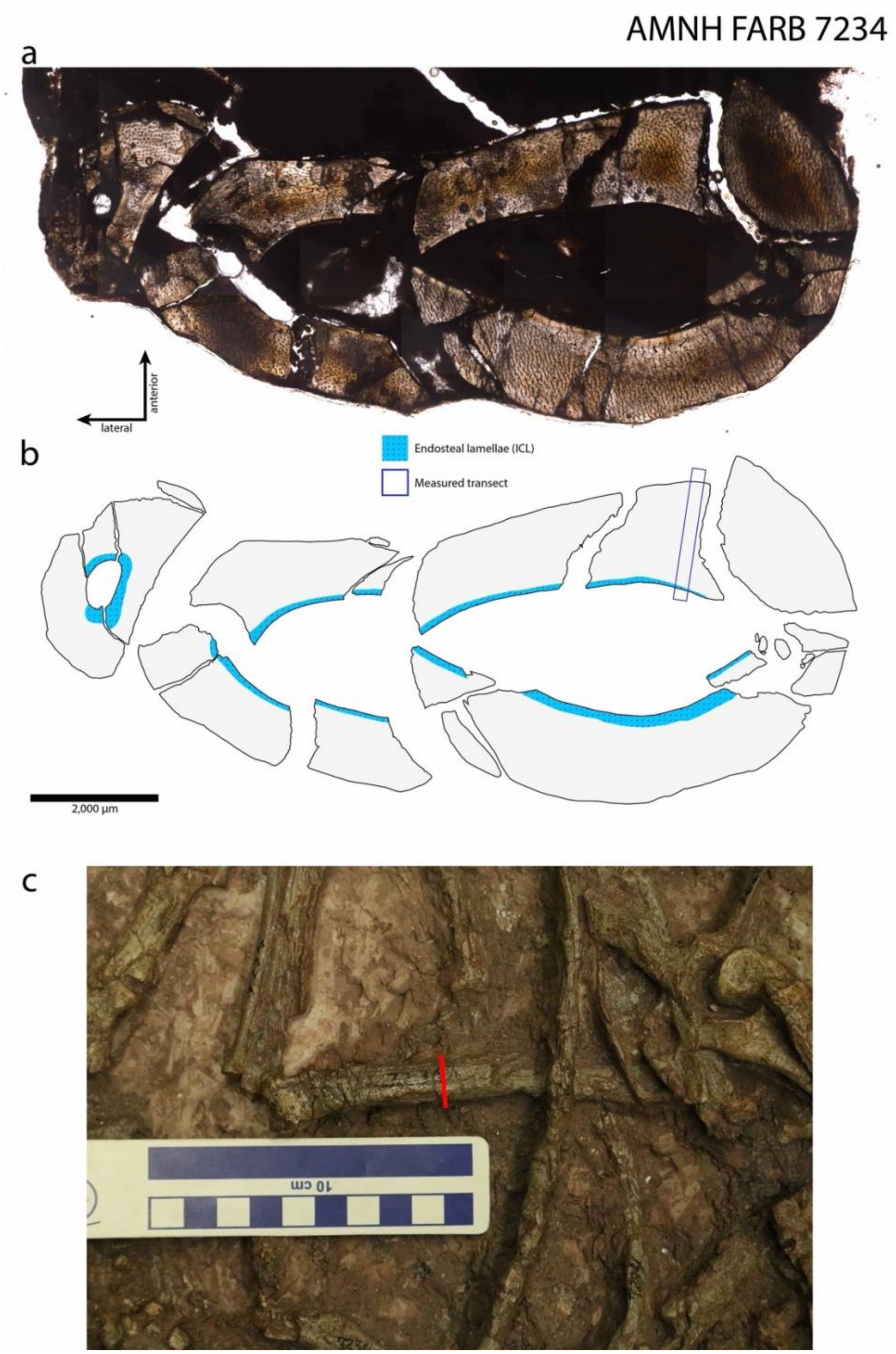

Supplementary Fig. 5 *Coelophysis bauri* left tibia and fibula AMNH FARB 7234. **a** Thin section photomicrograph. **b** Interpretive line drawing of **a**, indicating histological features and measured transect. **c** Sampling location for thin section indicated by red line. Scale bar for **a** and **b** is 2,000 µm. Each square on scale bar in **c** is 1 cm long.

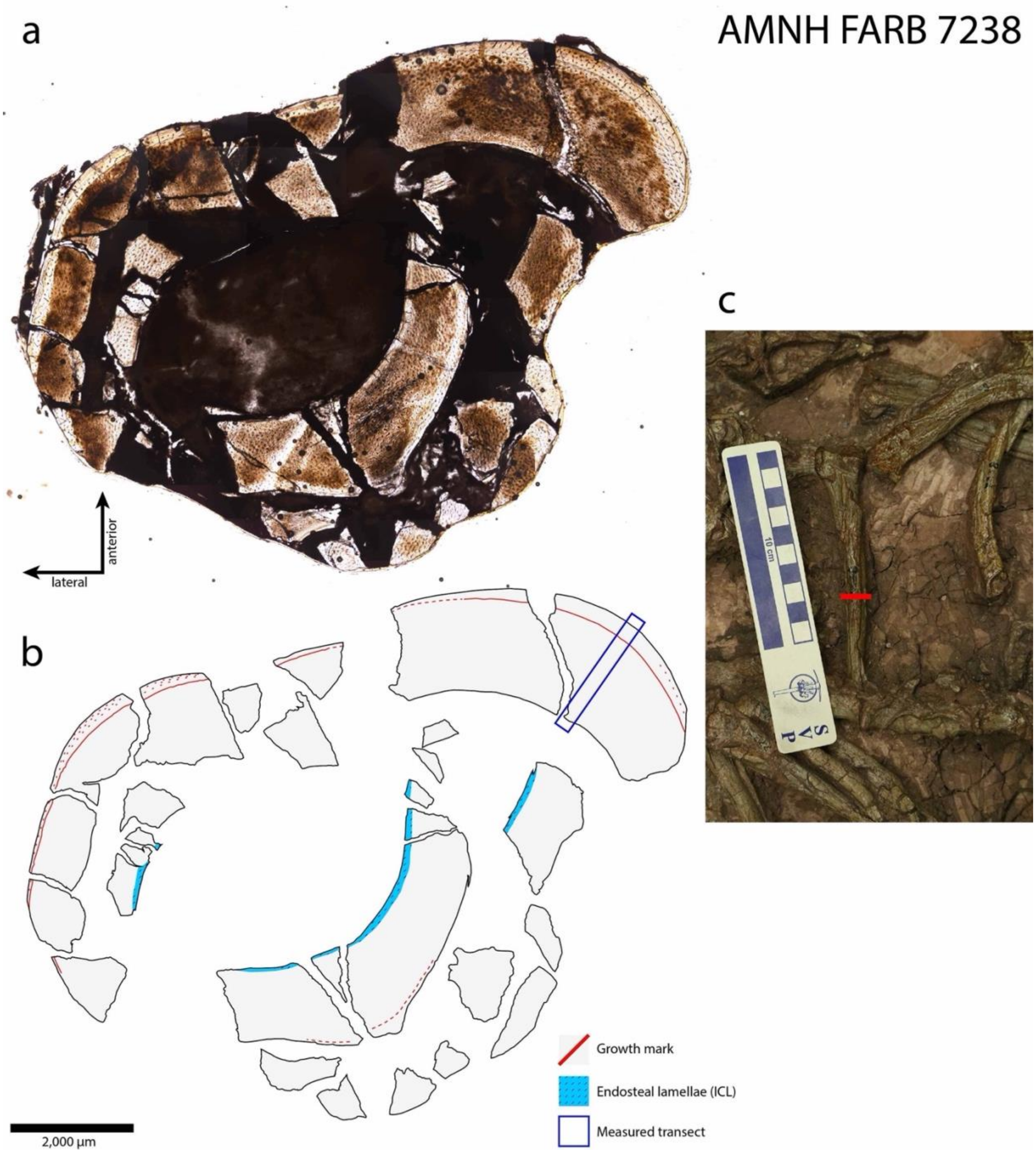

Supplementary Fig. 6 *Coelophysis bauri* left tibia AMNH FARB 7238. **a** Thin section photomicrograph. **b** Interpretive line drawing of **a**, indicating histological features and measured transect. **c** Sampling location for thin section indicated by red line. Scale bar for **a** and **b** is 2,000 µm. Each square on scale bar in **c** is 1 cm long.

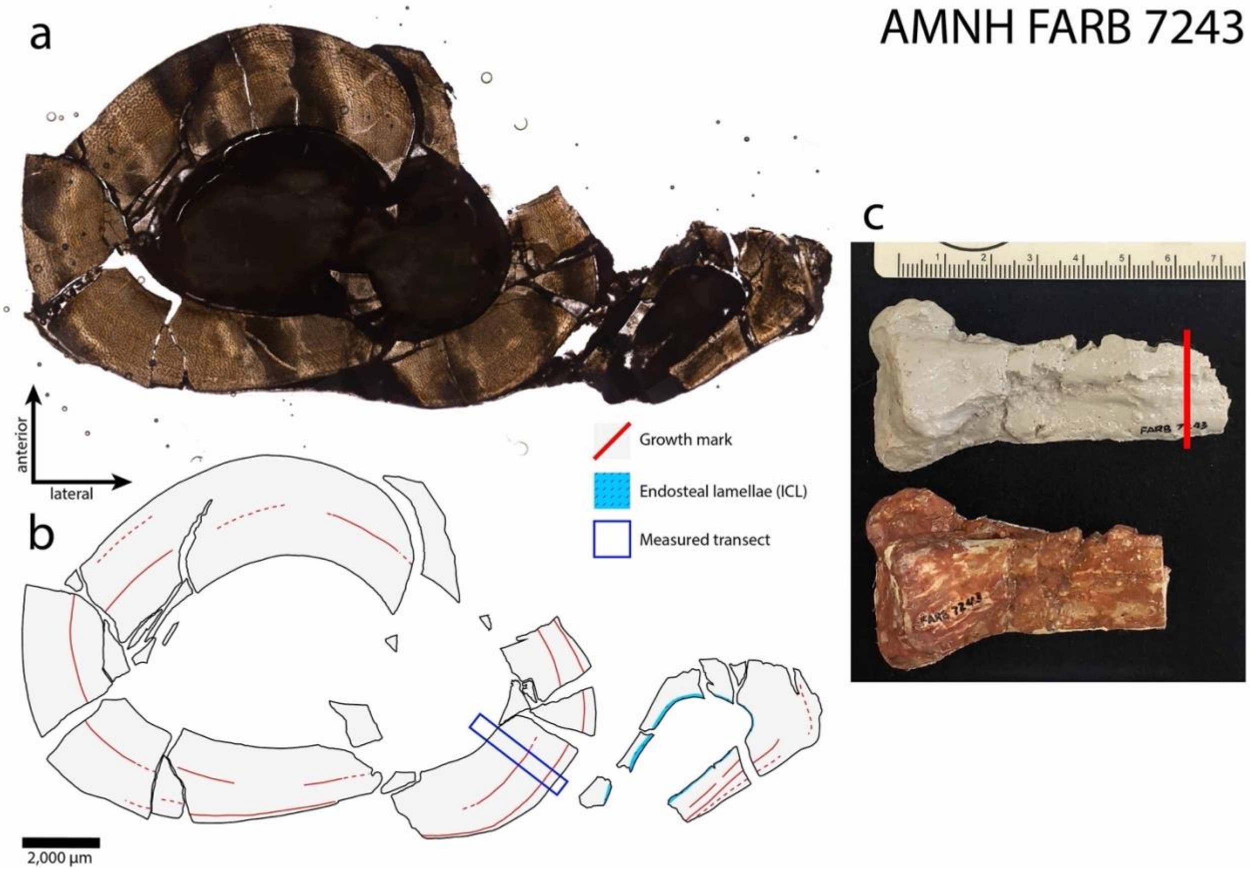

Supplementary Fig. 7 *Coelophysis bauri* right tibia and fibula AMNH FARB 7243. **a** Thin section photomicrograph. **b** Interpretive line drawing of **a**, indicating histological features and measured transect. **c** Sampling location for thin section indicated by red line. Scale bar for **a** and **b** is 2,000 µm. Each square on scale bar in **c** is 1 cm long. [Photo credit for **c**: C. Mehling, © 2021 American Museum of Natural History. All rights reserved.]

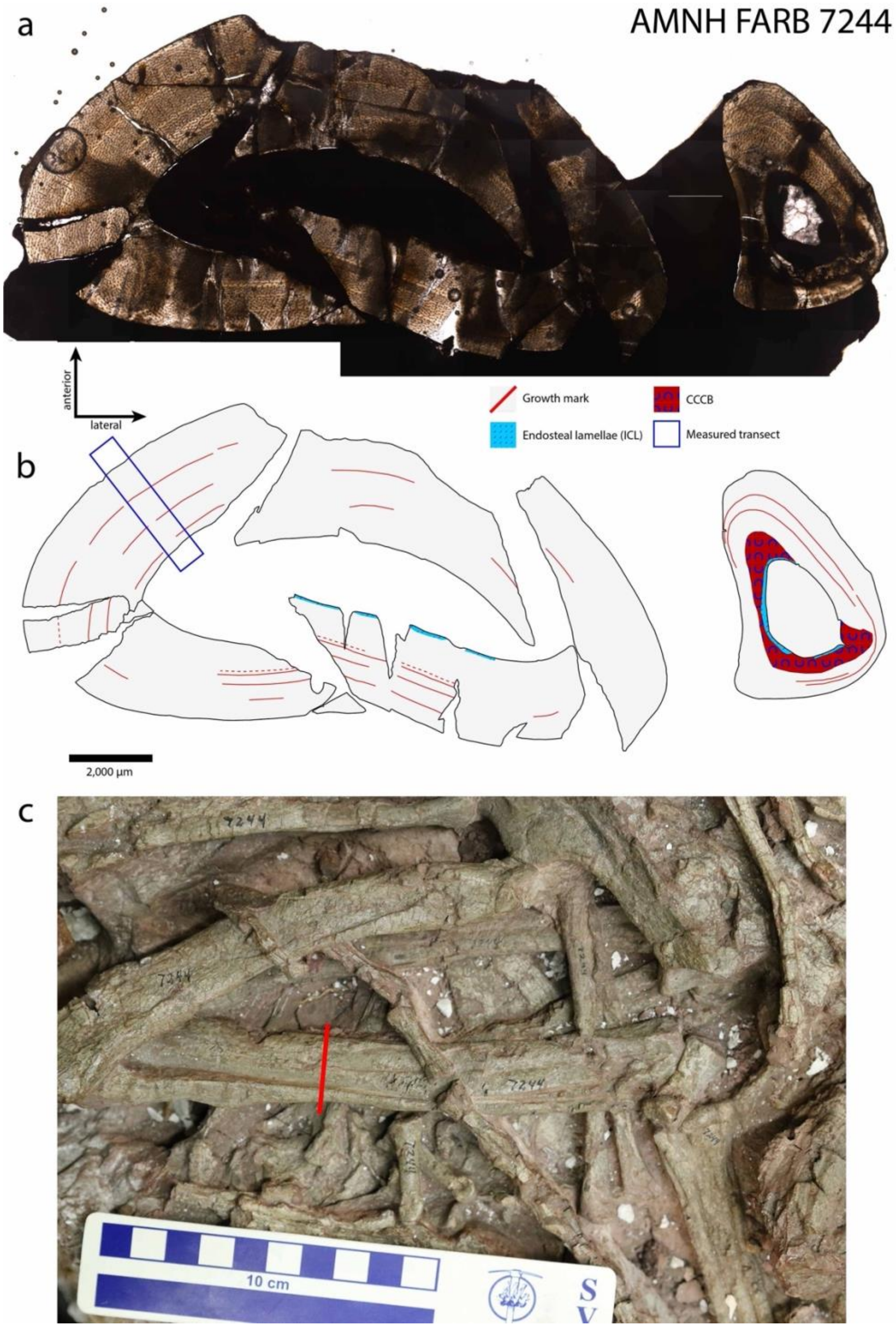

Supplementary Fig. 8 *Coelophysis bauri* left tibia and fibula AMNH FARB 7244. **a** Thin section photomicrograph. **b** Interpretive line drawing of **a**, indicating histological features and measured transect. **c** Sampling location for thin section indicated by red line. Scale bar for **a** and **b** is 2,000 µm. Each square on scale bar in **c** is 1 cm long.

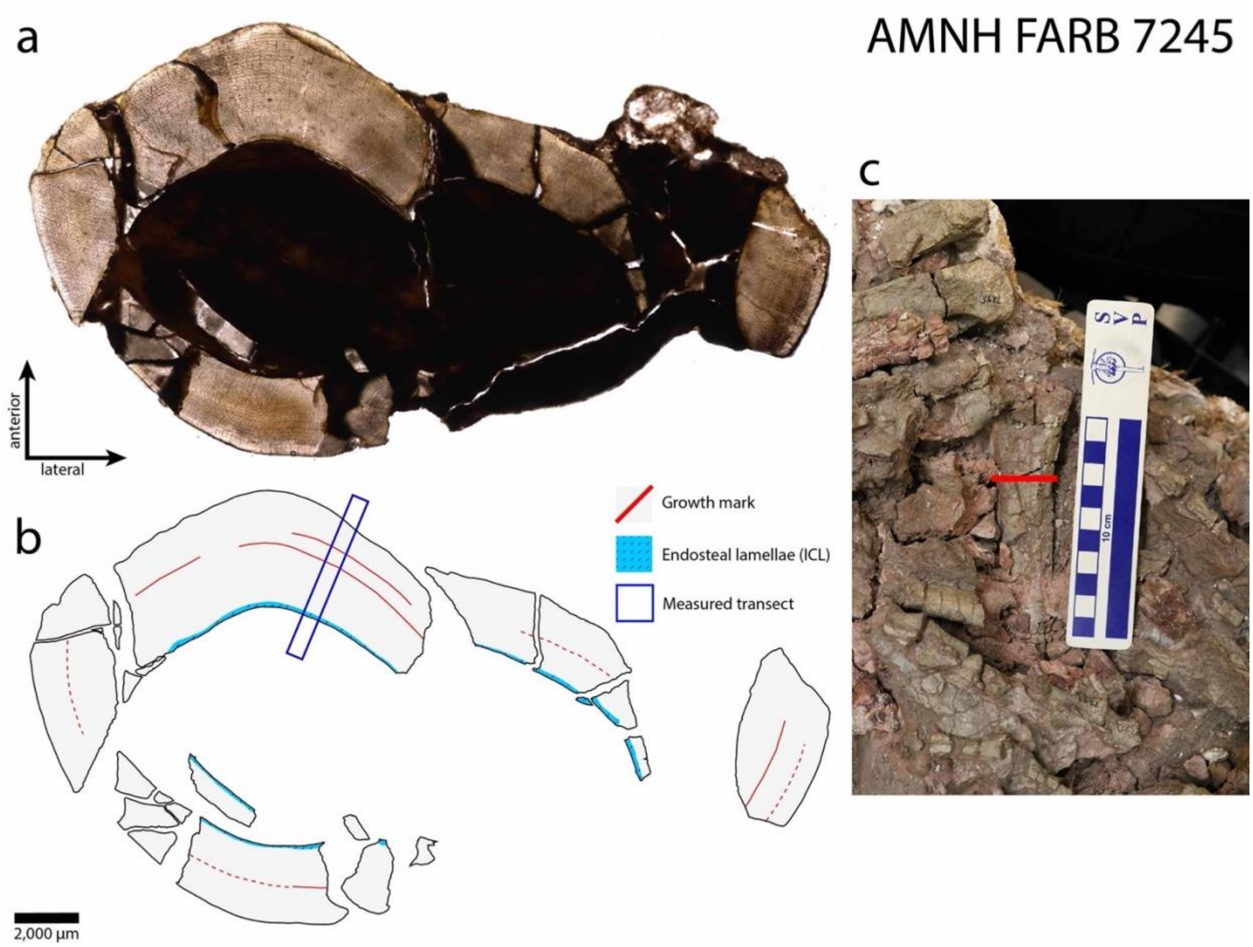

Supplementary Fig. 9 *Coelophysis bauri* right tibia AMNH FARB 7245. **a** Thin section photomicrograph. **b** Interpretive line drawing of **a**, indicating histological features and measured transect. **c** Sampling location for thin section indicated by red line. Scale bar for **a** and **b** is 2,000 μm. Each square on scale bar in **c** is 1 cm long.

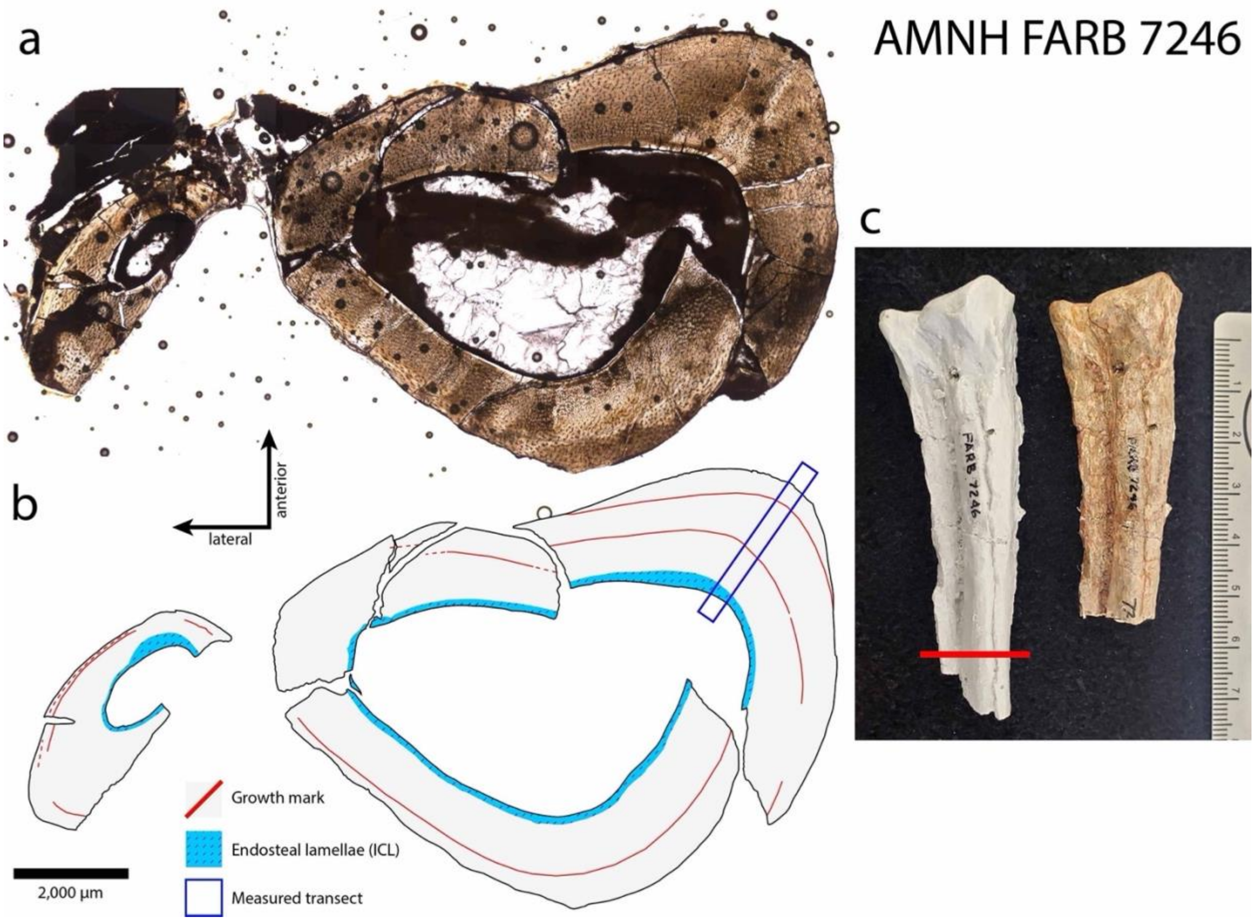

Supplementary Fig. 10 *Coelophysis bauri* right tibia and fibula AMNH FARB 7246. a Thin section photomicrograph. b Interpretive line drawing of a, indicating histological features and measured transect. c Sampling location for thin section indicated by red line. Scale bar for a and b is 2,000 µm. Each square on scale bar in c is 1 cm long. [Photo credit for c: C. Mehling, © 2021 American Museum of Natural History. All rights reserved.]

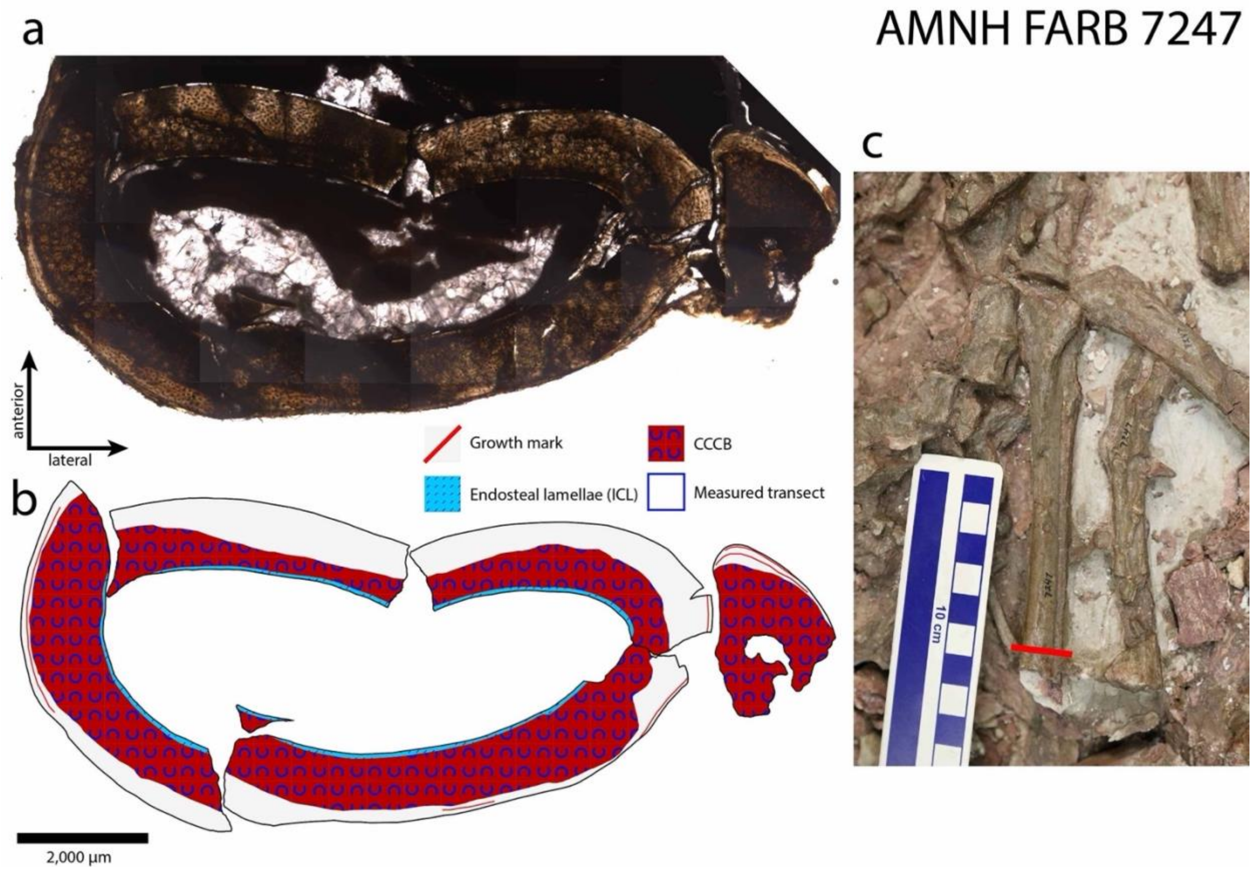

Supplementary Fig. 11 *Coelophysis bauri* left tibia and fibula AMNH FARB 7247. **a** Thin section photomicrograph. **b** Interpretive line drawing of **a**, indicating histological features and measured transect. **c** Sampling location for thin section indicated by red line. Scale bar for **a** and **b** is 2,000 µm. Each square on scale bar in **c** is 1 cm long.

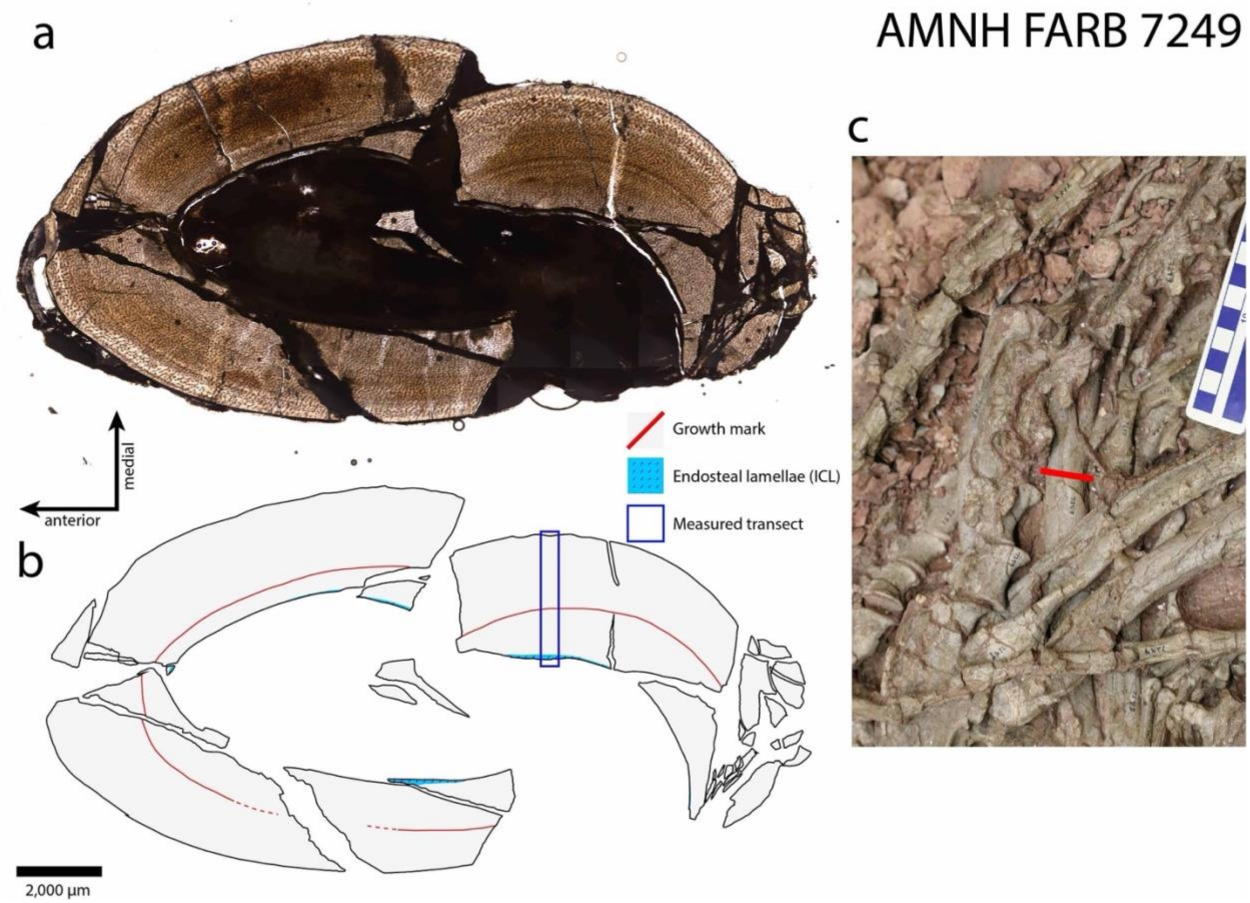

Supplementary Fig. 12 *Coelophysis bauri* left? tibia AMNH FARB 7249. **a** Thin section photomicrograph. **b** Interpretive line drawing of **a**, indicating histological features and measured transect. **c** Sampling location for thin section indicated by red line. Scale bar for **a** and **b** is 2,000 µm. Each square on scale bar in **c** is 1 cm long.

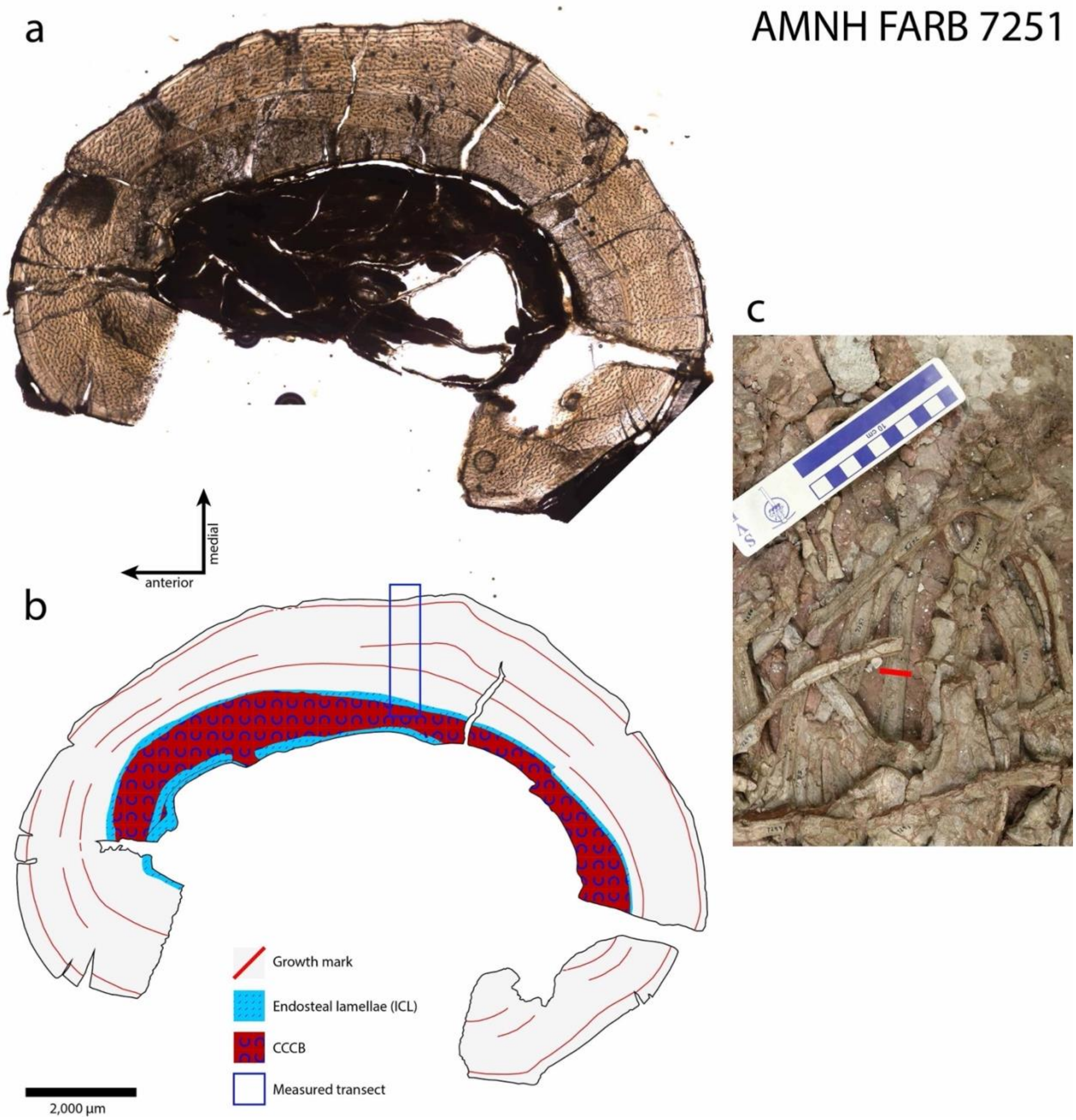

Supplementary Fig. 13 *Coelophysis bauri* left? tibia AMNH FARB 7251. **a** Thin section photomicrograph. **b** Interpretive line drawing of **a**, indicating histological features and measured transect. **c** Sampling location for thin section indicated by red line. Scale bar for **a** and **b** is 2,000 μm. Each square on scale bar in **c** is 1 cm long.

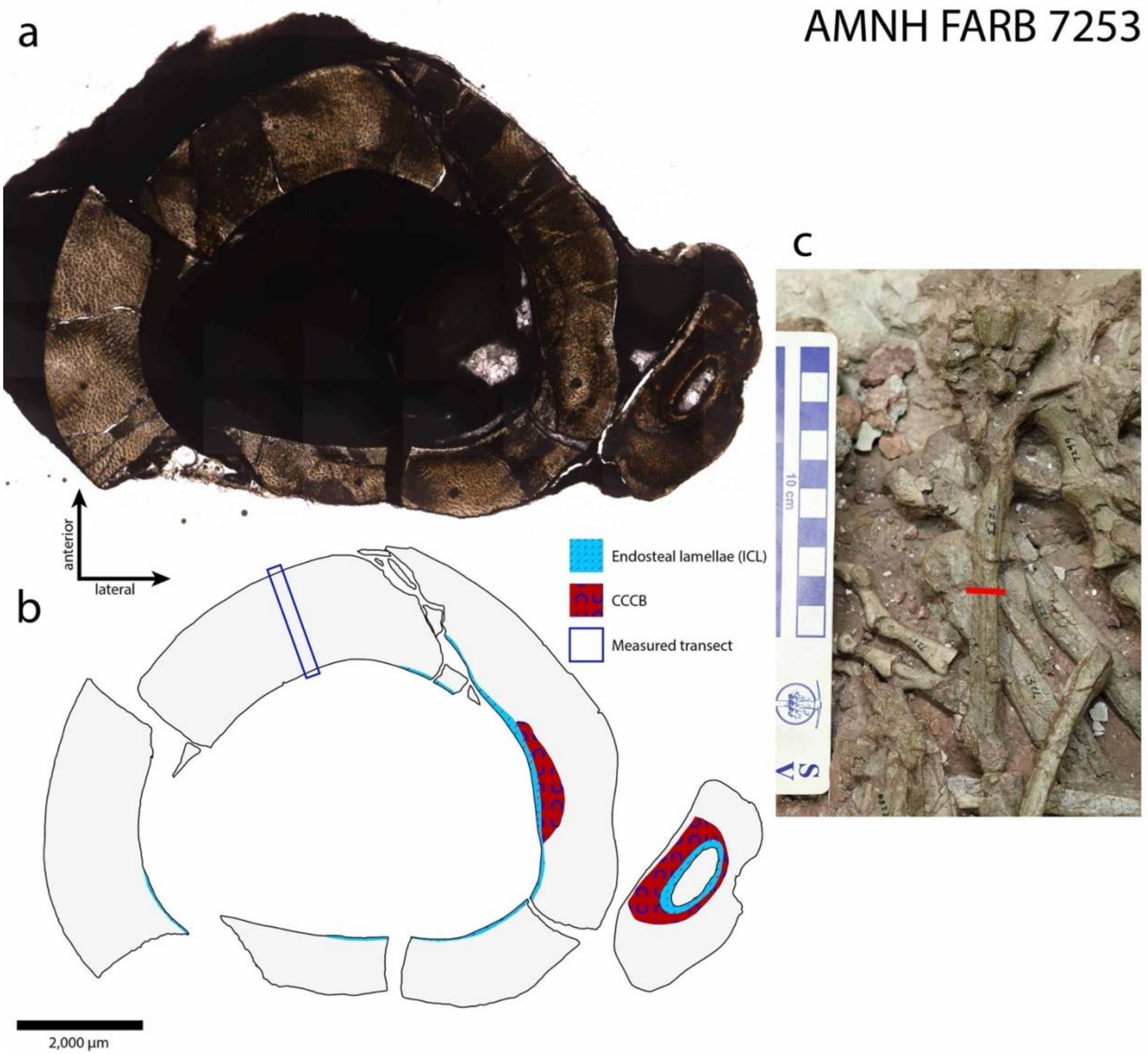

Supplementary Fig. 14 *Coelophysis bauri* right tibia and fibula AMNH FARB 7253. **a** Thin section photomicrograph. **b** Interpretive line drawing of **a**, indicating histological features and measured transect. **c** Sampling location for thin section indicated by red line. Scale bar for **a** and **b** is 2,000 μm. Each square on scale bar in **c** is 1 cm long.

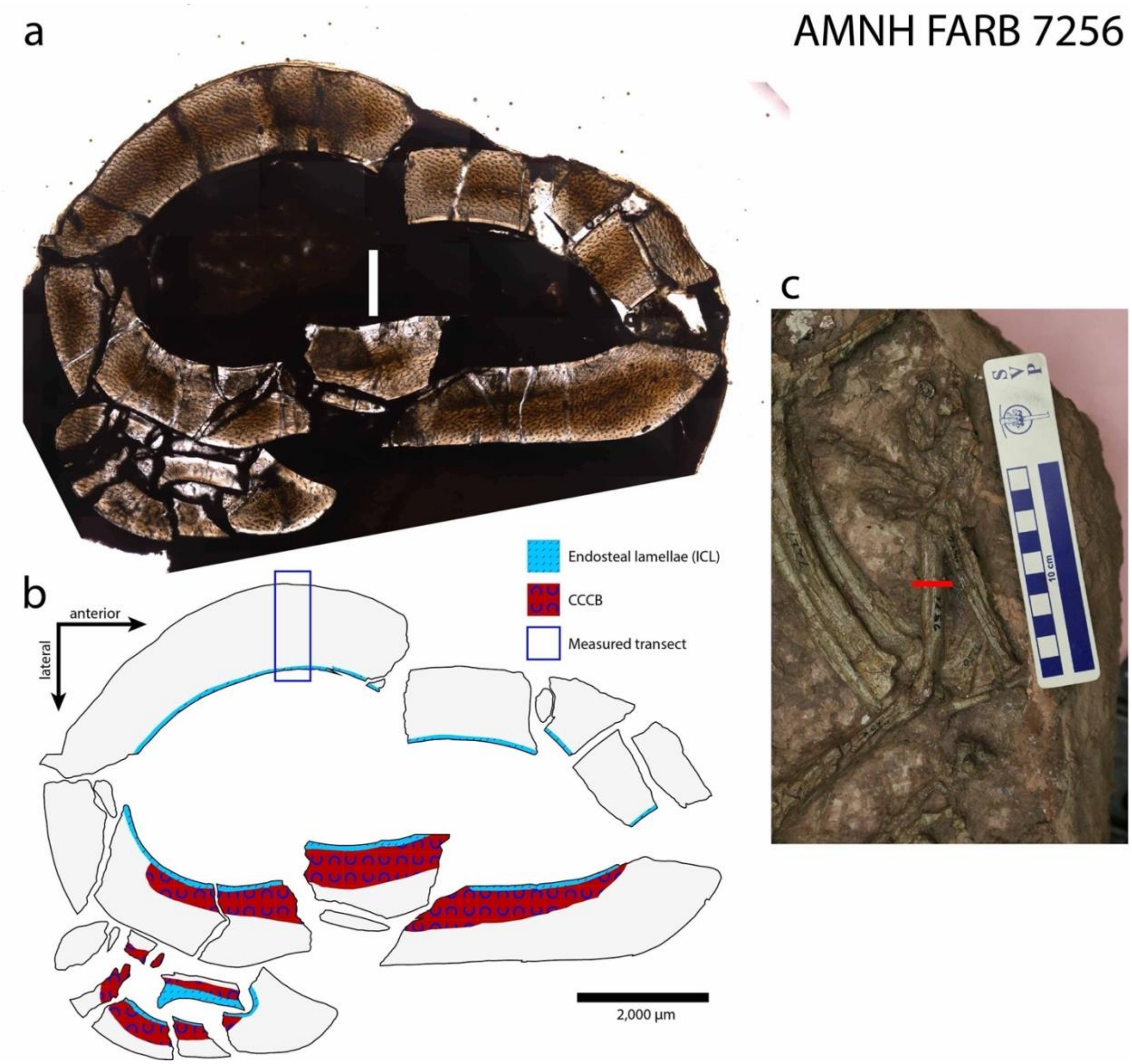

Supplementary Fig. 15 *Coelophysis bauri* right tibia and fibula AMNH FARB 7256. **a** Thin section photomicrograph. **b** Interpretive line drawing of **a**, indicating histological features and measured transect. **c** Sampling location for thin section indicated by red line. Scale bar for **a** and **b** is 2,000 µm. Each square on scale bar in **c** is 1 cm long.

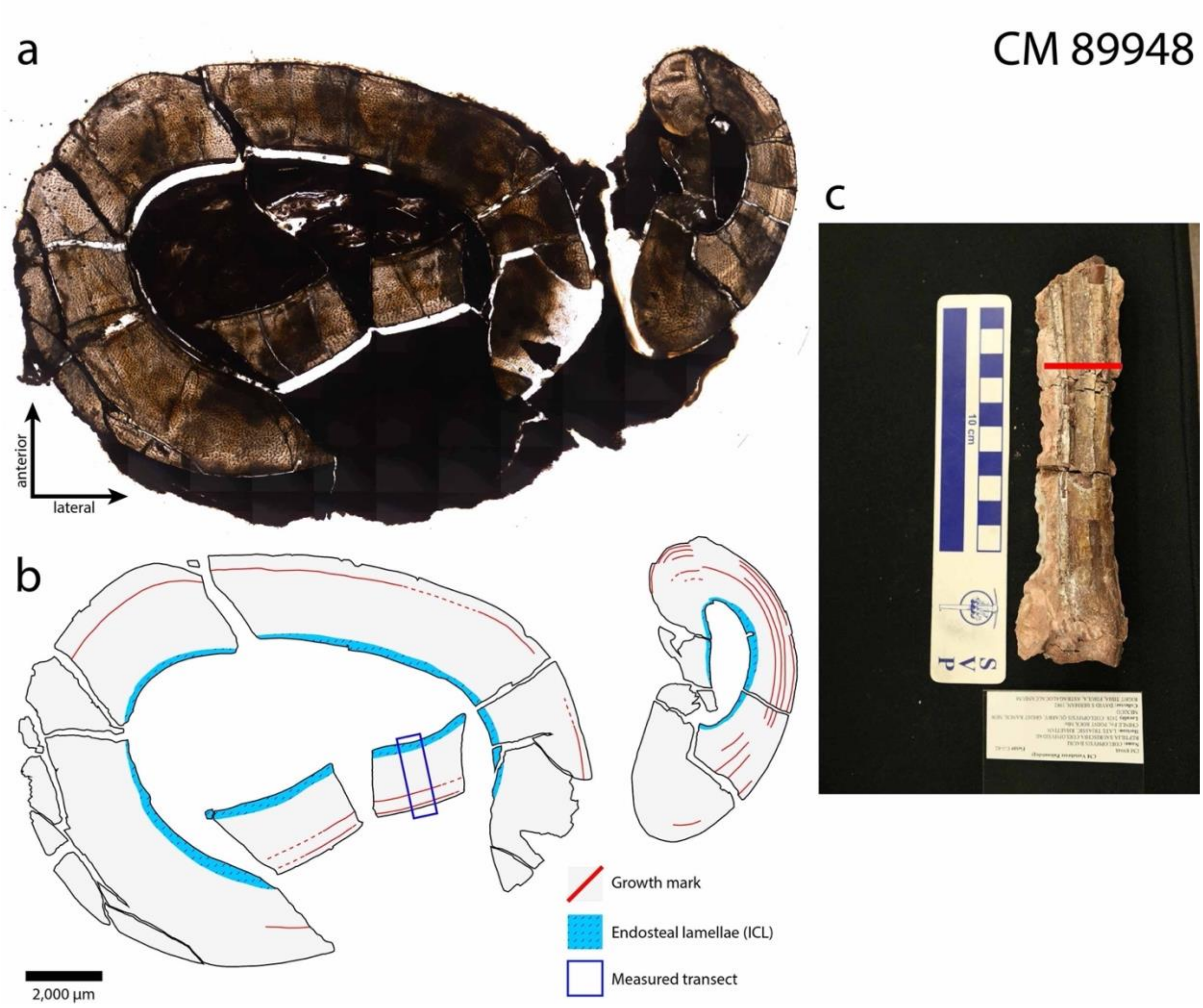

Supplementary Fig. 16 *Coelophysis bauri* right tibia and fibula CM 89948. **a** Thin section photomicrograph. **b** Interpretive line drawing of **a**, indicating histological features and measured transect. **c** Sampling location for thin section indicated by red line. Scale bar for **a** and **b** is 2,000 µm. Each square on scale bar in **c** is 1 cm long.

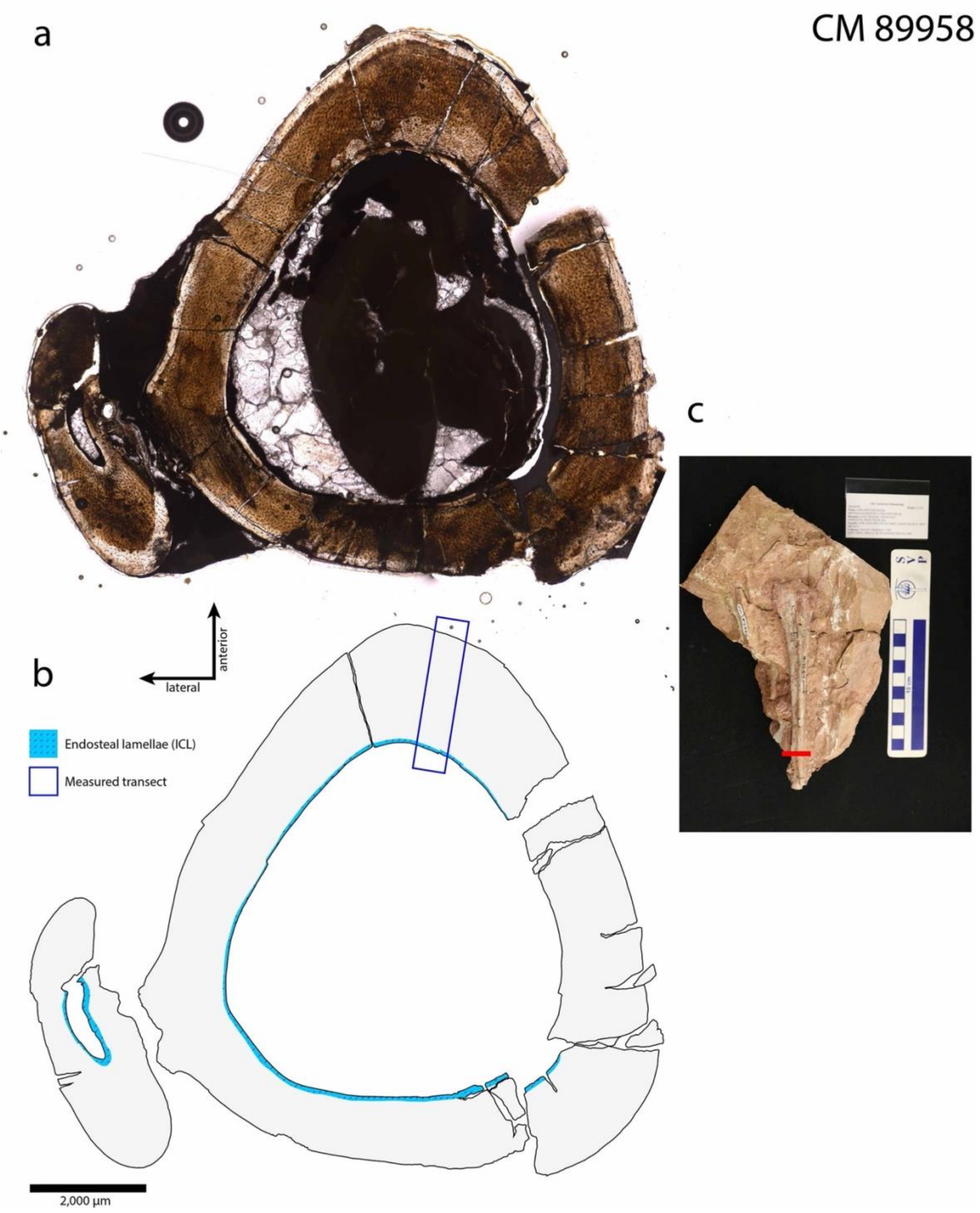

Supplementary Fig. 17 *Coelophysis bauri* left tibia and fibula CM 89958. **a** Thin section photomicrograph. **b** Interpretive line drawing of **a**, indicating histological features and measured transect. **c** Sampling location for thin section indicated by red line. Scale bar for **a** and **b** is 2,000 µm. Each square on scale bar in **c** is 1 cm long.

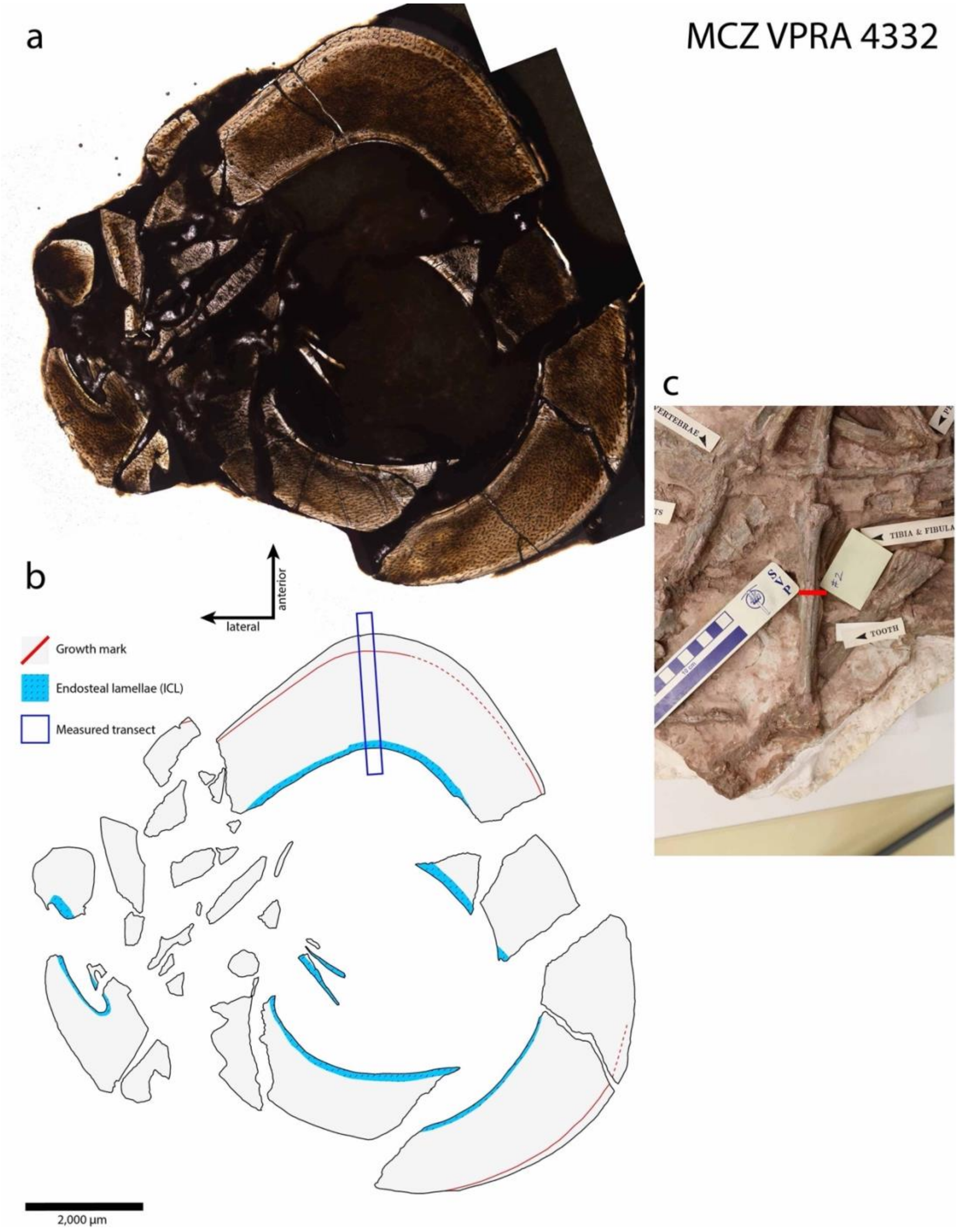

Supplementary Fig. 18 *Coelophysis bauri* left tibia and fibula MCZ VPRA 4332. **a** Thin section photomicrograph. **b** Interpretive line drawing of **a**, indicating histological features and measured transect. **c** Sampling location for thin section indicated by red line. Scale bar for **a** and **b** is 2,000 µm. Each square on scale bar in **c** is 1 cm long. [Photo credit: Museum of Comparative Zoology, Harvard University, © President and Fellows of Harvard College]

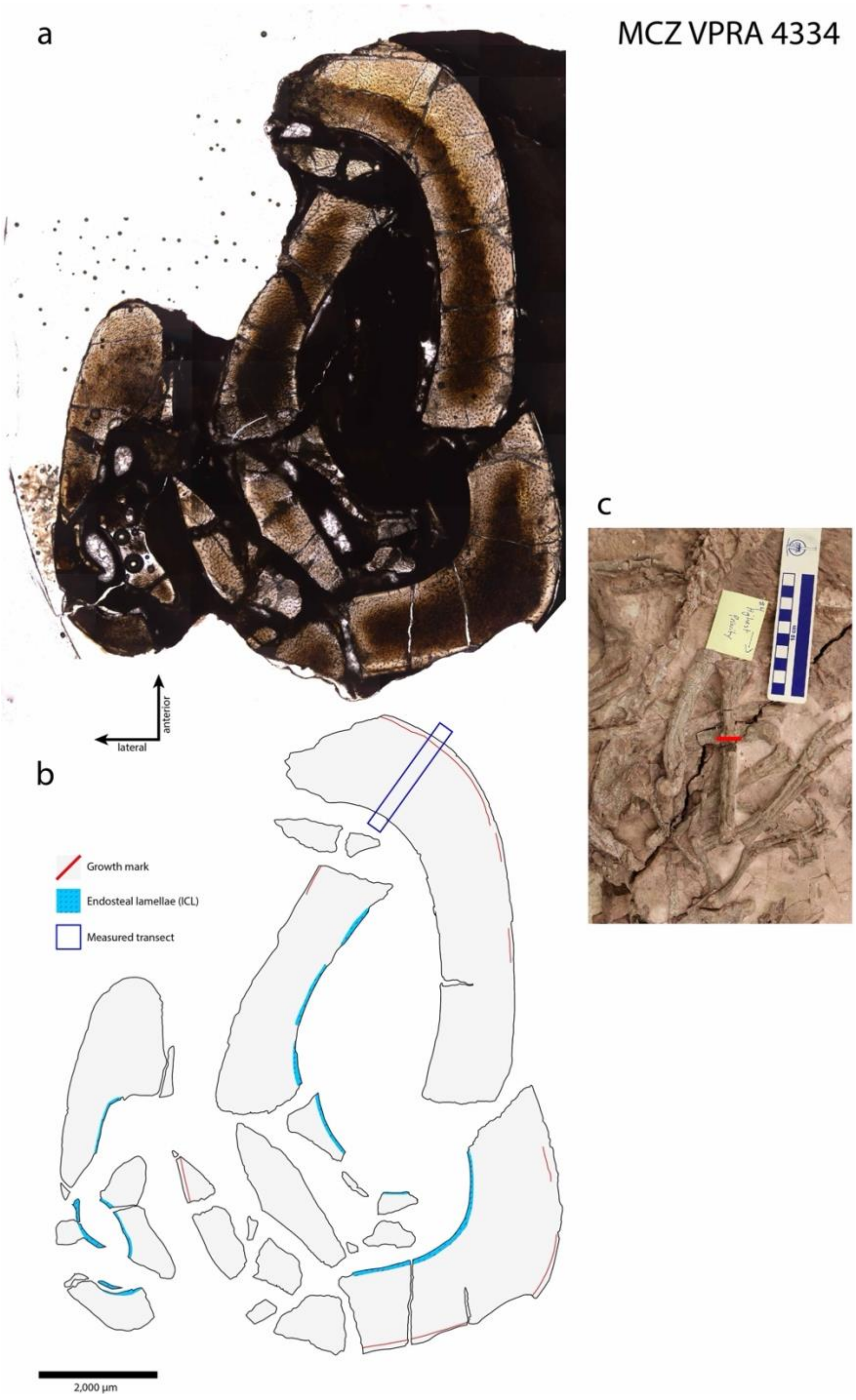

Supplementary Fig. 19 *Coelophysis bauri* right tibia and fibula MCZ VPRA 4334. **a** Thin section photomicrograph. **b** Interpretive line drawing of **a**, indicating histological features and measured transect. **c** Sampling location for thin section indicated by red line. Scale bar for **a** and **b** is 2,000 μm. Each square on scale bar in **c** is 1 cm long. [Photo credit: Museum of Comparative Zoology, Harvard University, © President and Fellows of Harvard College]

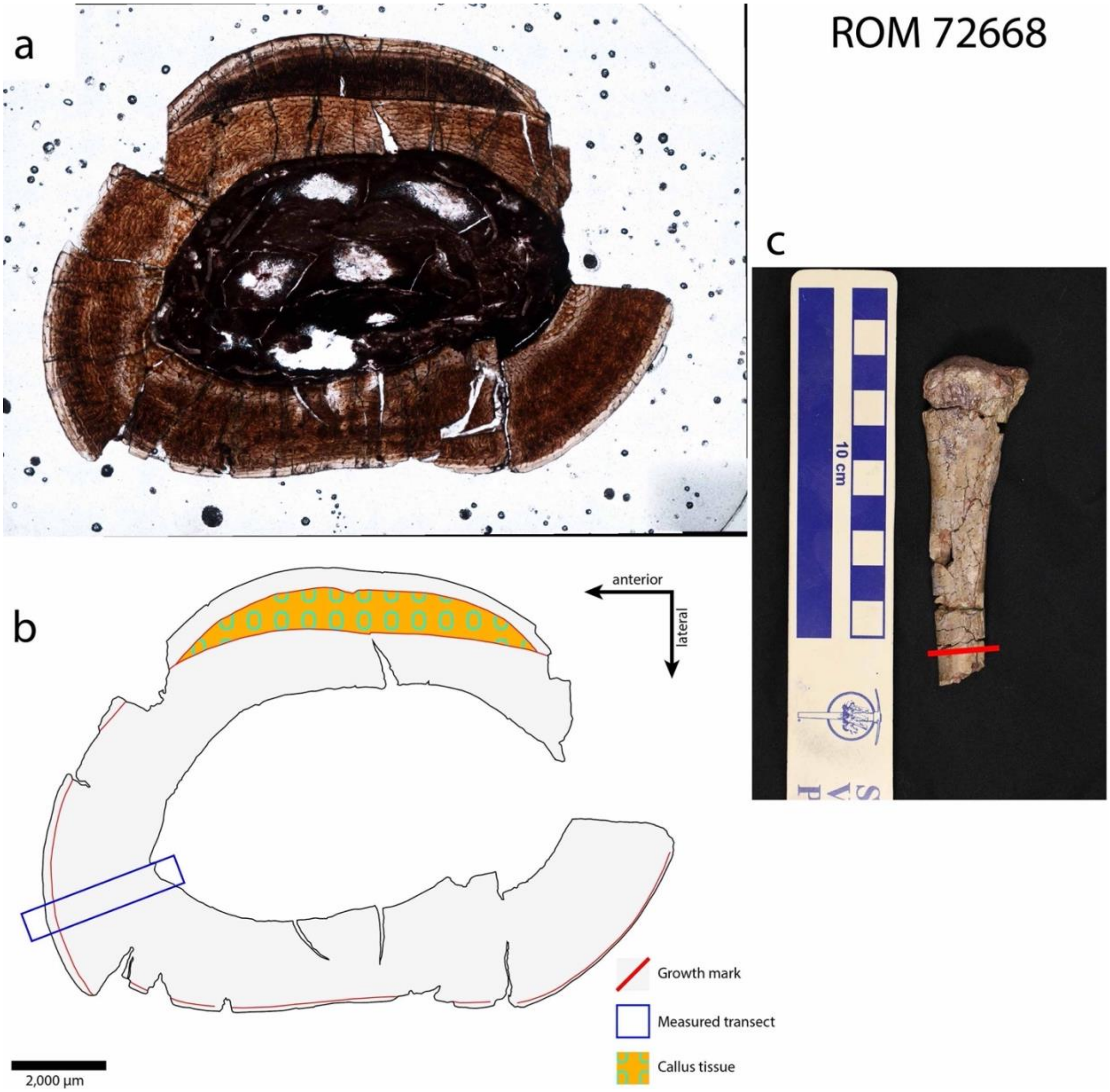

Supplementary Fig. 20 *Coelophysis bauri* right tibia ROM 72668. **a** Thin section photomicrograph. **b** Interpretive line drawing of **a**, indicating histological features and measured transect. **c** Sampling location for thin section indicated by red line. Scale bar for **a** and **b** is 2,000 µm. Each square on scale bar in **c** is 1 cm long. [Photo credit for **a**: D. J. Simon, University of Toronto and Royal Ontario Museum]

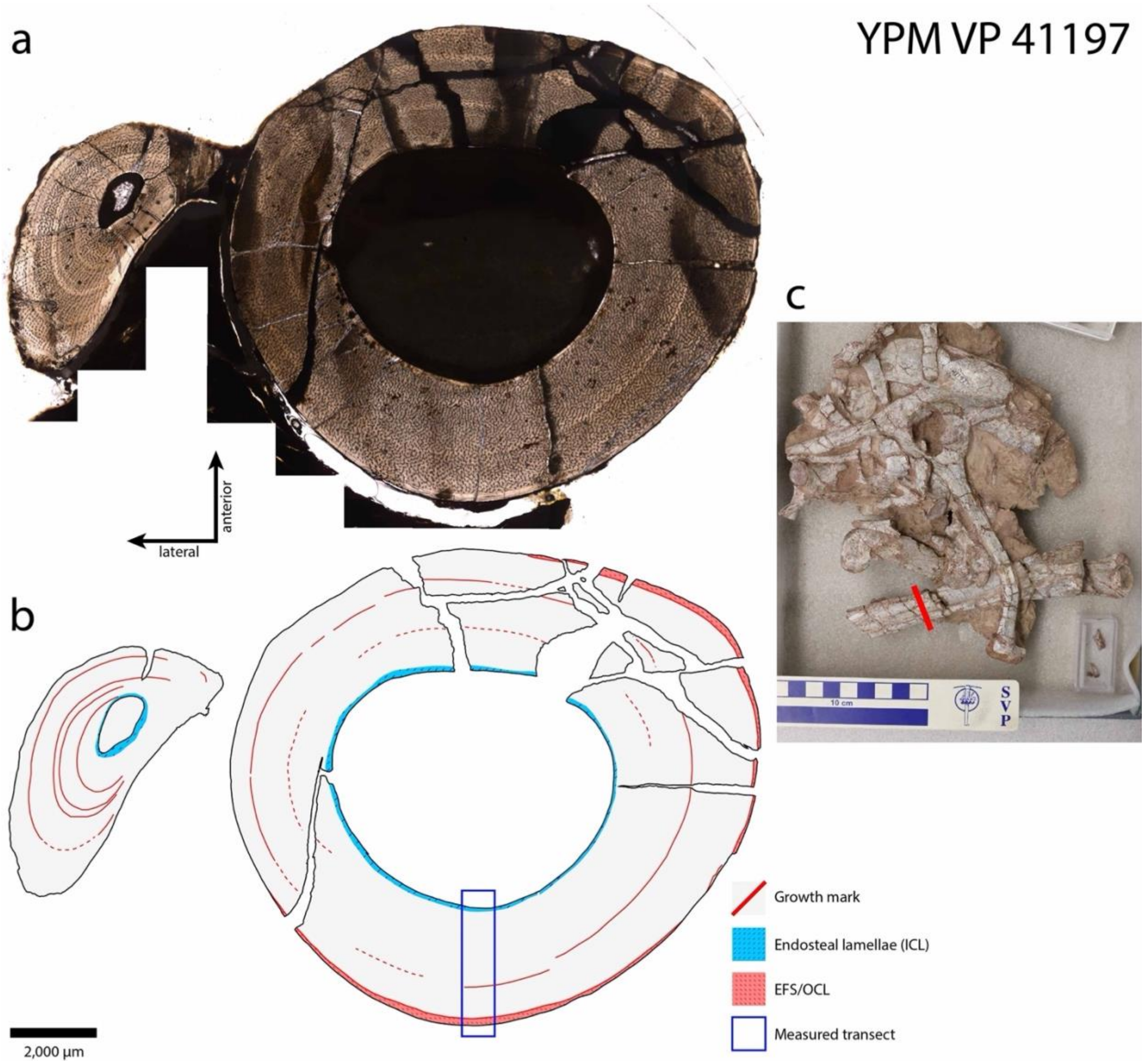

Supplementary Fig. 21 *Coelophysis bauri* left tibia and fibula YPM VP 41197. **a** Thin section photomicrograph. **b** Interpretive line drawing of **a**, indicating histological features and measured transect. **c** Sampling location for thin section indicated by red line. Scale bar for **a** and **b** is 2,000 µm. Each square on scale bar in **c** is 1 cm long.

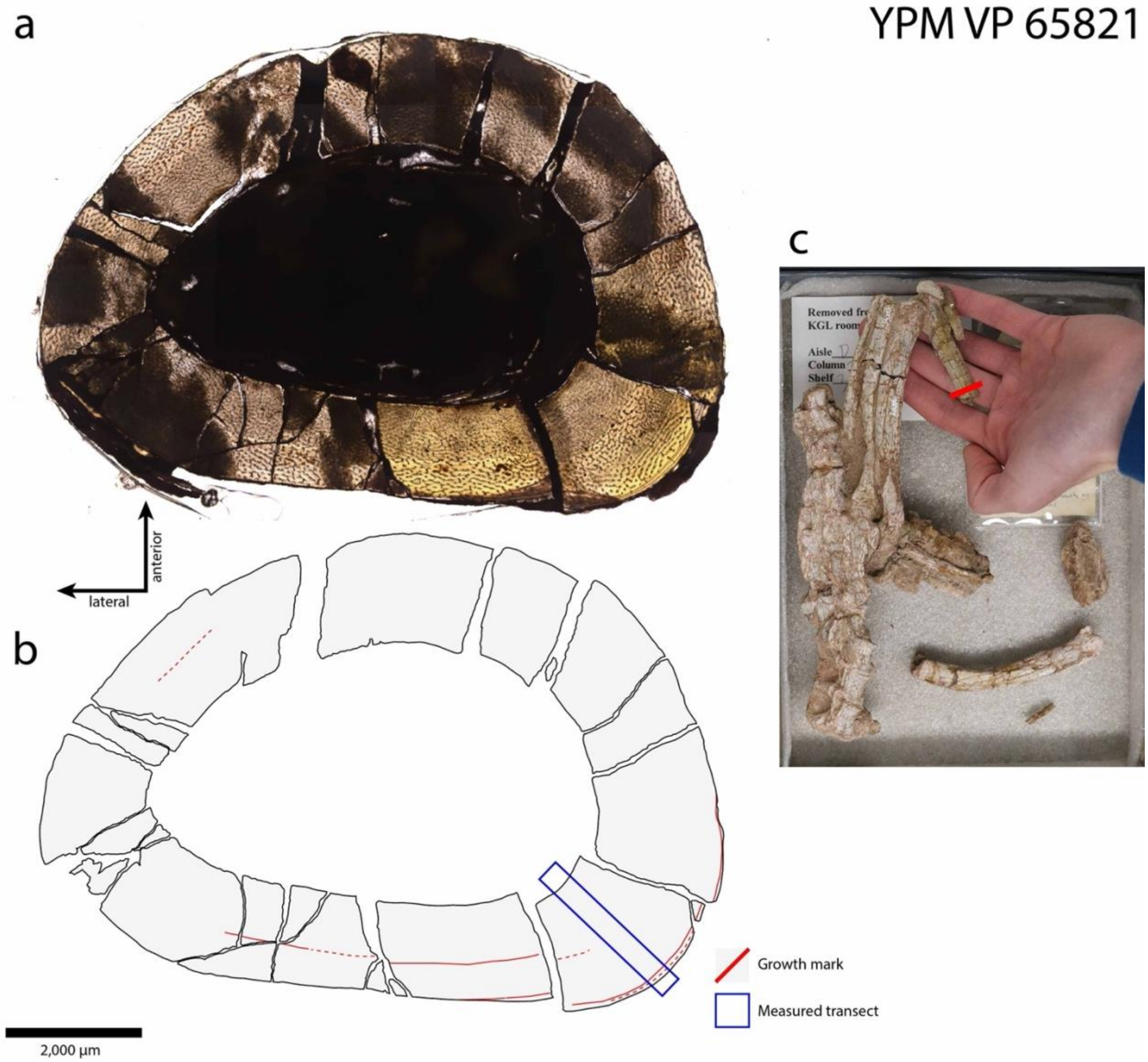

Supplementary Fig. 22 *Coelophysis bauri* right tibia YPM VP 65821. **a** Thin section photomicrograph. **b** Interpretive line drawing of **a**, indicating histological features and measured transect. **c** Sampling location for thin section indicated by red line. Scale bar for **a** and **b** is 2,000 µm.

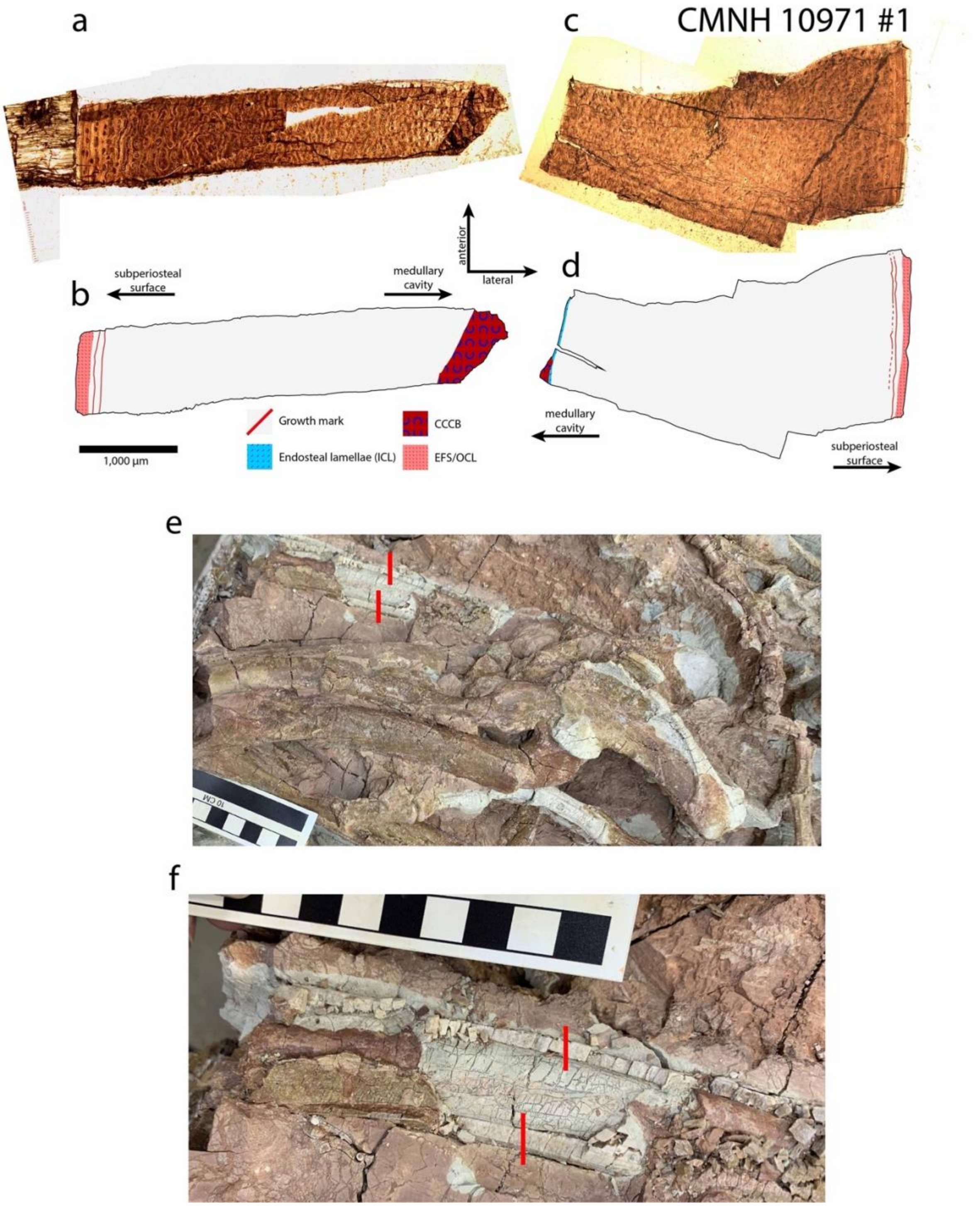

Supplementary Fig. 23 *Coelophysis bauri* right femur CMNH 10971 #1. **a** and **c** Thin section photomicrographs. **b** and **d** Interpretive line drawings of **a** and **c**, respectively, indicating histological features. **e** Sampling locations for thin sections indicated by red lines. **f** Closeup of **e**. Scale bar for **a**-**d** is 1,000 µm. Each square on scale bars in **e** and **f** is 1 cm long.

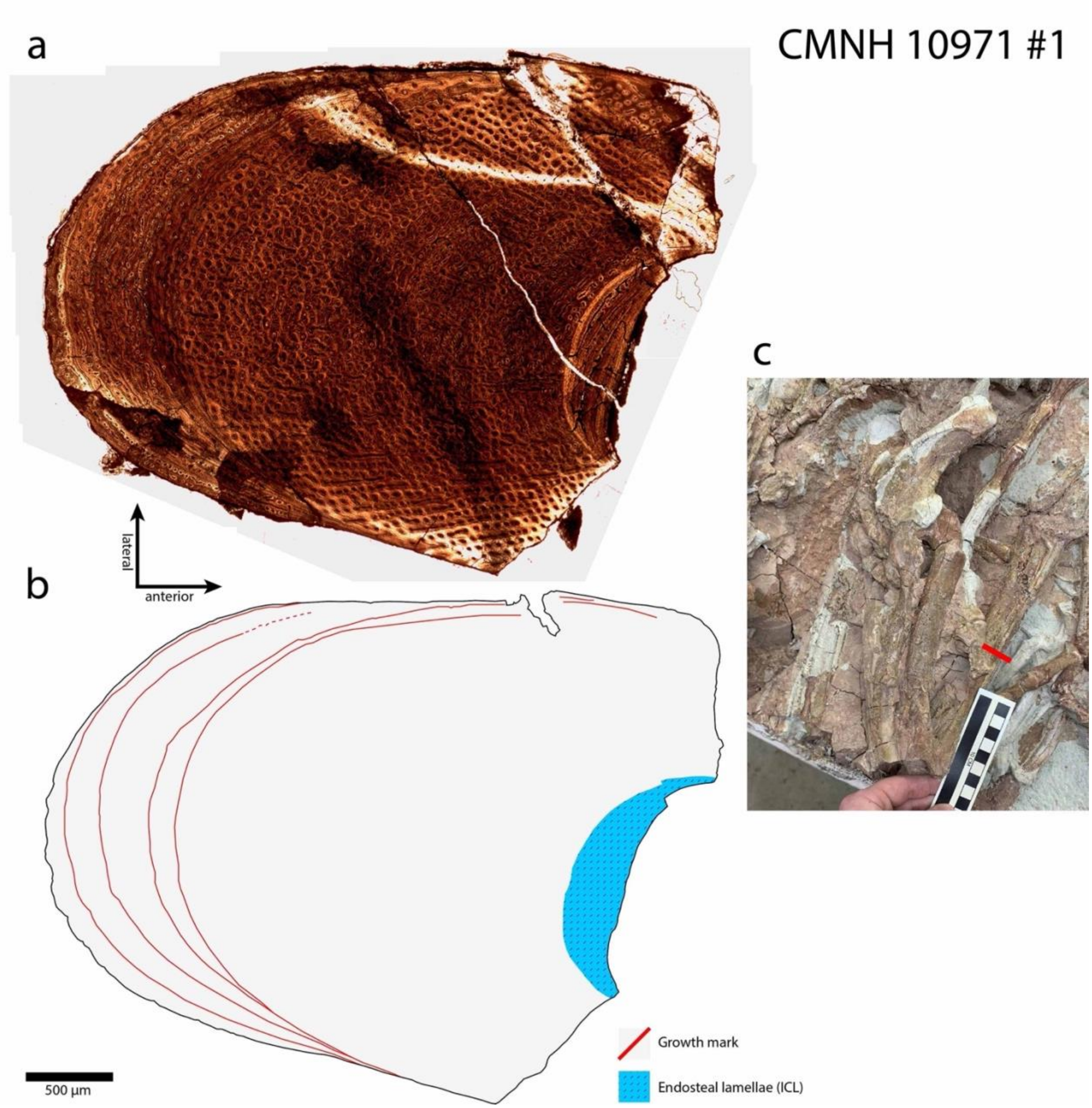

Supplementary Fig. 24 *Coelophysis bauri* left fibula CMNH 10971 #1. **a** Thin section photomicrograph. **b** Interpretive line drawing of **a**, indicating histological features. **c** Sampling location for thin section indicated by red line. Scale bar for **a** and **b** is 500 µm. Each square on scale bar in **c** is 1 cm long.

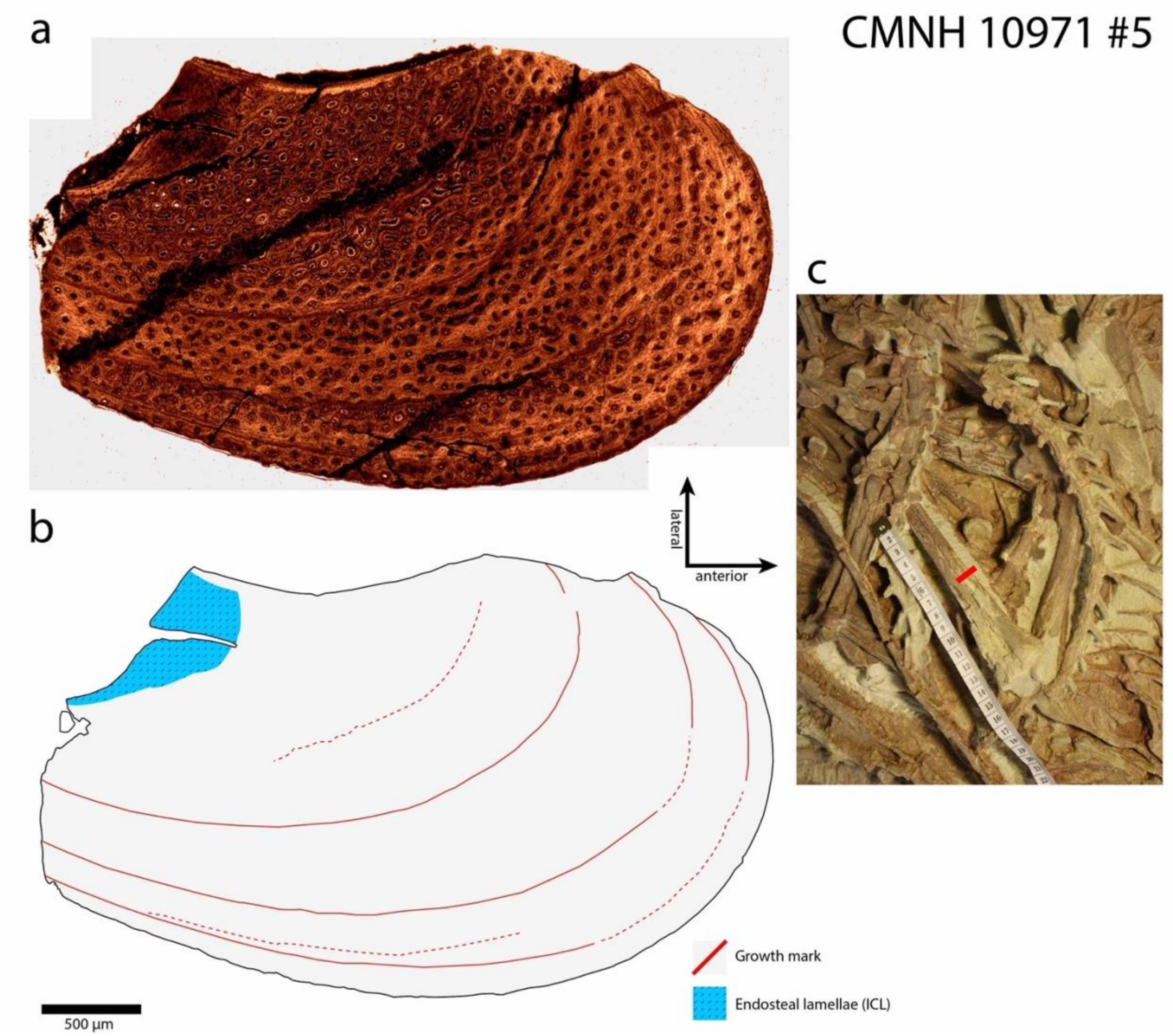

Supplementary Fig. 25 *Coelophysis bauri* right fibula CMNH 10971 #5. **a** Thin section photomicrograph. **b** Interpretive line drawing of **a**, indicating histological features. **c** Sampling location for thin section indicated by red line. Scale bar for **a** and **b** is 500 µm. Each tick mark on measuring tape in **c** is 1 cm long.

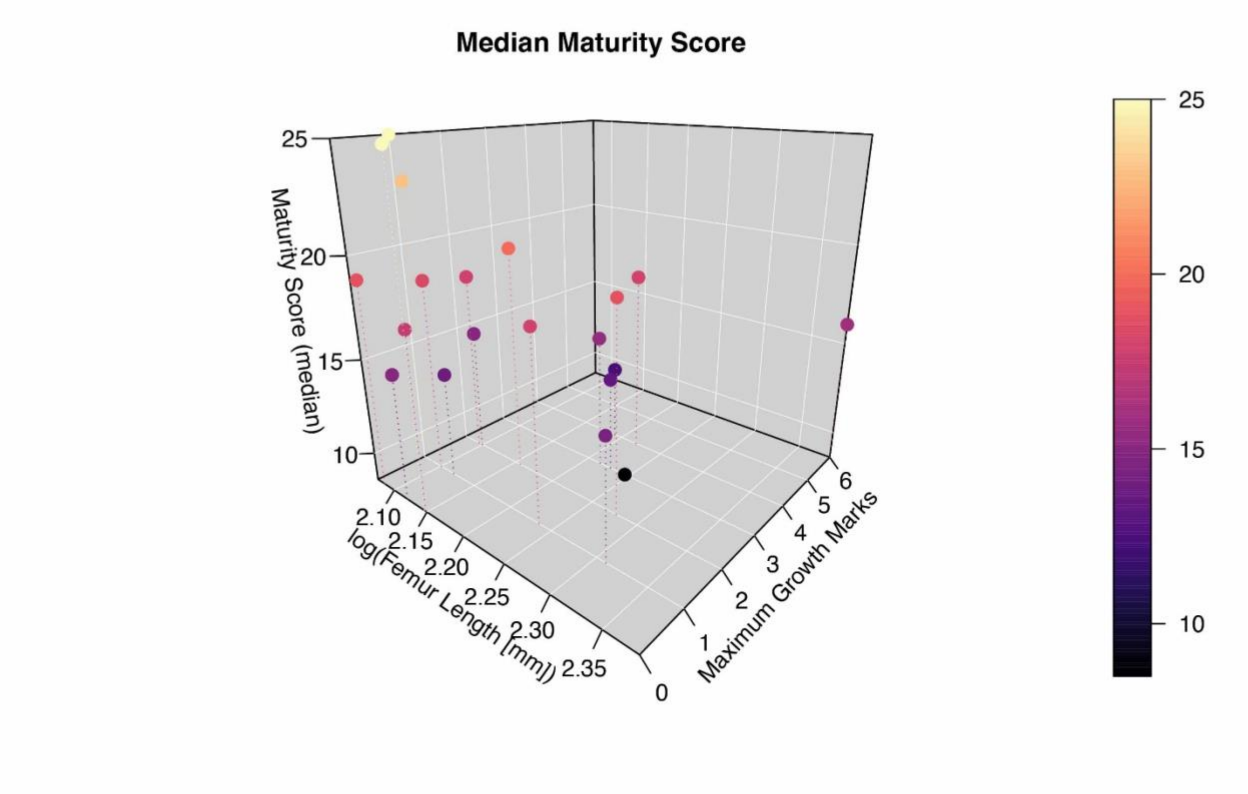

Supplementary Fig. 26 *Coelophysis bauri* log(femur length) vs. maximum growth mark count vs. median maturity score. Maturity scores symbolized by color scale.

Specimen	log(tibia circumference	Tibial growth marks	Fibular growth marks	Maximum growth marks	Tibia circumference (mm)	Femur length (mm)	log(femur length)	Minimum maturity score	Maximum maturity score	Median maturity score
AMNH FARB 7233	1.36865871	1	2	2	23.37	125.71	2.09936983	15	15	15
AMNH FARB 7253	1.37930552	0	0	0	23.95	139.98	2.14606599	25	25	25
AMNH FARB 7234	1.40088322	0	0	0	25.17	121.46	2.08443328	18	22	19
MCZ VPRA 4334	1.40619942	1	0	1	25.48	121.73	2.08539762	23	23	23
MCZ VPRA 4332	1.42160393	1	0	1	26.4	118.41	2.07338838	25	25	25
AMNH FARB 7246	1.42324587	2	1	2	26.5	124.3	2.09447113	18	18	18
AMNH FARB 7232	1.42683645	0	0	0	26.72	140.61	2.14801621	17	18	17.5
YPM VP 59865	1.43136376	1		1	27	131.8	2.11991541	9	19	14
AMNH FARB 7238	1.43584437	1		1	27.28	126	2.10037055	17	21	18.5
AMNH FARB 7256	1.44153804	0	0	0	27.64	132.12	2.12096857	14	19	15
CM 89958	1.47129171	0	0	0	29.6	120.68	2.0816353			
AMNH FARB 7229	1.48315921	0	0	0	30.42	132.24	2.12136284	15	15	15
AMNH FARB 7251	1.53970324	3		3	34.65	164	2.21484385	14	17	15.5
AMNH FARB 7228	1.54095481	1	1	1	34.75	172	2.23552845	18	18	18
AMNH FARB 7244	1.55266822	3	2	3	35.7	176	2.24551267	8	9	8.5
AMNH FARB 7249	1.55497346	1		1	35.89	204	2.30963017	14	15	14.5
YPM VP 41197	1.57829531	2	4	4	37.87	165.6	2.21906033	12	24	18
AMNH FARB 7243	1.59361831	2	2	2	39.23	189.41	2.2774029	10	28	19
CM 89948	1.610873	2	4	4	40.82	155.13	2.19069579	5	20	12.5
AMNH FARB 7245	1.66256897	2		2	45.98	143.37	2.15645829	12	28	20
ROM 72668	1.58069694	0		0	38.08					
CMNH 10971 #1			4	6		241.05	2.38210714	13	23	16
CMNH 10971 #5			3	3		169	2.22788671	7	15	13.5

Supplementary Table 1 Measurements, growth mark counts, and maturity scores (see main text) for all analyzed *Coelophysis bauri* specimens. We also provide these data in .csv format (Supplementary Data 1) to run with R code (Supplementary Data 2).

Supplementary Table 2 (separate file) Growth zone thicknesses for *Coelophysis bauri* specimens.

Supplementary Table 3 (separate file) Growth zone thicknesses for *Alligator mississippiensis* specimens.

Supplementary Table 4 (separate file) Growth zone thicknesses for *Maiasaura peeblesorum* specimens. Data from[2], except measurements marked with an asterisk, which were newly measured from the original photomicrographs following the methods of Woodward et al.[2].

Supplementary Data 1 (separate file) Data file in .csv format containing all data for running quantitative analyses using R code (Supplementary Data 2).

Supplementary Data 2 (separate file) R code to be used with .csv file (Supplementary Data 1) for running quantitative analyses.

Supplementary References

1. Wells, J. W. Coral growth and geochronometry. *Nature*. **197**, 948–950 (1963).
2. Woodward, H. N., Freedman Fowler, E. A., Farlow, J. O. & Horner, J. R. *Maiasaura*, a model organism for extinct vertebrate population biology: a large sample statistical assessment of growth dynamics and survivorship. *Paleobiology*. **41**, 503–527 (2015).
3. de Margerie, E., Cubo, J. & Castanet, J. Bone typology and growth rate: testing and quantifying ‘Amprino’s rule’ in the mallard (*Anas platyrhynchos*). *Comptes Rendus Biologies*. **325**, 221–230 (2002).
4. Padian, K., de Ricqlès, A. J. & Horner, J. R. Dinosaurian growth rates and bird origins. *Nature*. **412**, 405–408 (2001).
5. Padian, K., Horner, J. R. & Ricqlès, A. D. Growth in small dinosaurs and pterosaurs: the evolution of archosaurian growth strategies. *J. Vertebr. Paleontol.* **24**, 555–571 (2004).
6. Horner, J. R. & Padian, K. Age and growth dynamics of *Tyrannosaurus rex*. *Proc. R. Soc. Lond. B Biol. Sci.* **271**, 1875–1880 (2004).
