# Supplementary material for: Osteohistology of a Triassic dinosaur population reveals highly variable growth trajectories typified early dinosaur ontogeny
Source: Sci Rep. 2022 Oct 15;12:17321. doi: 10.1038/s41598-022-22216-x (PMC9569331; doi:10.1038/s41598-022-22216-x)
Supplement: Supplementary file 6 — Supplementary Information 6. [file 41598_2022_22216_MOESM6_ESM.docx]

#install.packages("ggplot2")

#install.packages("plot3D")

#install.packages("viridis")

#setwd()

#Call up packages

library(ggplot2)

library(plot3D)

library(viridis)

#.CSV file "Coelophysis_data_for_R.csv" contains data for all code.

#The following are explanations for all variable names:

#Specimen -> specimen number

#logcir -> the log of the tibia circumference

#tibgm -> tibial growth marks

#fibgm -> fibular growth marks

#maxgm -> maximum growth mark number (whether tibia or fibula)

#tibcir -> tibia circumference

#femlen -> femur length

#logfem -> log of the femur length

#matscore.lwr -> the minimum of the estimated maturity score range

#matscore.upr -> the maximum of the estimated maturity score range

#matscore.med -> the median of the estimated maturity score range

##### Regressions #####

#Read in data file

coelo<-read.csv("Coelophysis_data_for_R.csv")

## Tibia growth marks (not logged) ##

#Linear regression, all data

line<-lm(coelo$tibgm~coelo$tibcir) #Linear regression of log tibia circumference and tibia growth marks

summary(line)

#Poisson regression, all data

poisson<-glm(coelo$tibgm~coelo$tibcir, family = "poisson") #Poisson regression of log tibia circumference and tibia growth marks

summary(poisson)

pseudoR2 <- 1-(poisson$deviance/poisson$null.deviance); pseudoR2 #Pseudo R-squared

pchisq(poisson$deviance, df=poisson$df.residual, lower.tail=FALSE) #Deviance goodness of fit test

#Plot

ggp <- ggplot(data=coelo, aes(tibcir, tibgm)) +

theme_bw() +

theme(legend.position = c(0.2,0.7))+

xlab("Tibia Circumference (mm)")+

ylab("Tibia Growth Marks")+

geom_point() +

scale_y_continuous(breaks = seq(0, 5, by = 1)) +

geom_smooth(method = "glm",

method.args = list(family = "poisson"),

alpha = 0.08, fill = "red",

aes(color = "red")) +

stat_smooth(method = "lm",

fill = "blue", alpha = 0.08,

aes(color = "blue")) +

scale_color_identity(name = "Model fit",

breaks = c("red", "blue"),

labels = c("Poisson", "Linear"),

guide = "legend"); ggp

ggsave("Regressions_TibiaGMs_notLogged_withROM.eps", device=cairo_ps, fallback_resolution = 600)

##Tibia growth marks, without ROM specimen

(not logged)

#Removing ROM specimen

coelo2<-coelo[1:20,]

#Linear regression, all data

line<-lm(coelo2$tibgm~coelo2$tibcir) #Linear regression of log tibia circumference and tibia growth marks

summary(line)

#Poisson regression, all data

poisson<-glm(coelo2$tibgm~coelo2$tibcir, family = "poisson") #Poisson regression of log tibia circumference and tibia growth marks

summary(poisson)

pseudoR2 <- 1-(poisson$deviance/poisson$null.deviance); pseudoR2 #Pseudo R-squared

pchisq(poisson$deviance, df=poisson$df.residual, lower.tail=FALSE) #Deviance goodness of fit test

#Plot

ggp <- ggplot(data=coelo2, aes(tibcir, tibgm)) +

theme_bw() +

theme(legend.position = c(0.2,0.7))+

xlab("Tibia Circumference (mm)")+

ylab("Tibia Growth Marks")+

geom_point() +

scale_y_continuous(breaks = seq(0, 5, by = 1)) +

geom_smooth(method = "glm",

method.args = list(family = "poisson"),

alpha = 0.08, fill = "red",

aes(color = "red")) +

stat_smooth(method = "lm",

fill = "blue", alpha = 0.08,

aes(color = "blue")) +

scale_color_identity(name = "Model fit",

breaks = c("red", "blue"),

labels = c("Poisson", "Linear"),

guide = "legend"); ggp

ggsave("Regressions_TibiaGMs_notLogged_withoutROM.eps", device=cairo_ps, fallback_resolution = 600)

##Tibia growth marks (logged)

#Linear regression, all data

line<-lm(coelo$tibgm~coelo$logcir) #Linear regression of log tibia circumference and tibia growth marks

summary(line)

#Poisson regression, all data

poisson<-glm(coelo$tibgm~coelo$logcir, family = "poisson") #Poisson regression of log tibia circumference and tibia growth marks

summary(poisson)

pseudoR2 <- 1-(poisson$deviance/poisson$null.deviance); pseudoR2 #Pseudo R-squared

pchisq(poisson$deviance, df=poisson$df.residual, lower.tail=FALSE) #Deviance goodness of fit test

#Plot

ggp <- ggplot(data=coelo, aes(logcir, tibgm)) +

theme_bw() +

theme(legend.position = c(0.2,0.7))+

xlab("log(Tibia Circumference")+

ylab("Tibia Growth Marks")+

geom_point() +

scale_y_continuous(breaks = seq(0, 5, by = 1)) +

geom_smooth(method = "glm",

method.args = list(family = "poisson"),

alpha = 0.08, fill = "red",

aes(color = "red")) +

stat_smooth(method = "lm",

fill = "blue", alpha = 0.08,

aes(color = "blue")) +

scale_color_identity(name = "Model fit",

breaks = c("red", "blue"),

labels = c("Poisson", "Linear"),

guide = "legend"); ggp

ggsave("Regressions_TibiaGMs_withROM.eps", device=cairo_ps, fallback_resolution = 600)

#Removing ROM specimen

coelo2<-coelo[1:20,]

#Linear regression

line<-lm(coelo2$tibgm~coelo2$logcir) #Linear regression of log tibia circumference and tibia growth marks

summary(line)

#Poisson regression

poisson<-glm(coelo2$tibgm~coelo2$logcir, family = "poisson") #Poisson regression of log tibia circumference and tibia growth marks

summary(poisson)

poisson

pseudoR2 <- 1-(poisson$deviance/poisson$null.deviance); pseudoR2 #Pseudo R-squared

pchisq(poisson$deviance, df=poisson$df.residual, lower.tail=FALSE) #Deviance goodness of fit test

#Plot

ggp <- ggplot(data=coelo2, aes(logcir, tibgm)) +

theme_bw() +

theme(legend.position = c(0.2,0.7))+

xlab("log(Tibia Circumference)")+

ylab("Tibia Growth Marks")+

geom_point() +

scale_y_continuous(breaks = seq(0, 5, by = 1)) +

geom_smooth(method = "glm",

method.args = list(family = "poisson"),

alpha = 0.08, fill = "red",

aes(color = "red")) +

stat_smooth(method = "lm",

fill = "blue", alpha = 0.08,

aes(color = "blue")) +

scale_color_identity(name = "Model fit",

breaks = c("red", "blue"),

labels = c("Poisson", "Linear"),

guide = "legend"); ggp

ggsave("Regressions_TibiaGMs_withoutROM.eps", device=cairo_ps, fallback_resolution = 600)

##Fibula growth marks

coelo3 <- na.omit(coelo[1:21, c(1,2,4)]) #Omit specimens without fibulae

#Linear regression

line<-lm(coelo3$fibgm~coelo3$logcir) #Linear regression of log tibia circumference and fibula growth marks

summary(line)

#Poisson regression

poisson<-glm(coelo3$fibgm~coelo3$logcir, family = "poisson") #Poisson regression of log tibia circumference and fibula growth marks

summary(poisson)

pseudoR2 <- 1-(poisson$deviance/poisson$null.deviance); pseudoR2 #Pseudo R-squared

pchisq(poisson$deviance, df=poisson$df.residual, lower.tail=FALSE) #Deviance goodness of fit test

#Plot

ggp <- ggplot(data=coelo3, aes(logcir, fibgm)) +

theme_bw() +

theme(legend.position = c(0.2,0.7))+

xlab("log(Tibia Circumference)")+

ylab("Fibula Growth Marks")+

geom_point() +

scale_y_continuous(breaks = seq(0, 5, by = 1)) +

geom_smooth(method = "glm",

method.args = list(family = "poisson"),

alpha = 0.08, fill = "red",

aes(color = "red")) +

stat_smooth(method = "lm",

fill = "blue", alpha = 0.08,

aes(color = "blue")) +

scale_color_identity(name = "Model fit",

breaks = c("red", "blue"),

labels = c("Poisson", "Linear"),

guide = "legend"); ggp

ggsave("Regressions_FibulaGMs.eps", device=cairo_ps, fallback_resolution = 600)

##Maximum growth marks

#Linear regression, all data

line<-lm(coelo$maxgm~coelo$logcir) #Linear regression of log tibia circumference and maximum number of growth marks

summary(line)

#Poisson regression, all data

poisson<-glm(coelo$maxgm~coelo$logcir, family = "poisson") #Poisson regression of log tibia circumference and maximum number of growth marks

summary(poisson)

pseudoR2 <- 1-(poisson$deviance/poisson$null.deviance); pseudoR2 #Pseudo R-squared

pchisq(poisson$deviance, df=poisson$df.residual, lower.tail=FALSE) #Deviance goodness of fit test

#Plot

ggp <- ggplot(data=coelo, aes(logcir, maxgm)) +

theme_bw() +

theme(legend.position = c(0.2,0.7))+

xlab("log(Tibia Circumference)")+

ylab("Maximum Growth Marks")+

geom_point() +

scale_y_continuous(breaks = seq(0, 5, by = 1)) +

geom_smooth(method = "glm",

method.args = list(family = "poisson"),

alpha = 0.08, fill = "red",

aes(color = "red")) +

stat_smooth(method = "lm",

fill = "blue", alpha = 0.08,

aes(color = "blue")) +

scale_color_identity(name = "Model fit",

breaks = c("red", "blue"),

labels = c("Poisson", "Linear"),

guide = "legend"); ggp

ggsave("Regressions_MaxGMs_withROM.eps", device=cairo_ps, fallback_resolution = 600)

#Removing ROM specimen

coelo2<-coelo[1:20,]

#Linear regression

line<-lm(coel2o$maxgm~coelo2$logcir) #Linear regression of log tibia circumference and maximum number of growth marks

summary(line)

#Poisson regression

poisson<-glm(coelo2$maxgm~coelo2$logcir, family = "poisson") #Poisson regression of log tibia circumference and maximum number of growth marks

summary(poisson)

poisson

pseudoR2 <- 1-(poisson$deviance/poisson$null.deviance); pseudoR2 #Pseudo R-squared

pchisq(poisson$deviance, df=poisson$df.residual, lower.tail=FALSE) #Deviance goodness of fit test

#Plot

ggp <- ggplot(data=coelo2, aes(logcir, maxgm)) +

theme_bw() +

theme(legend.position = c(0.2,0.7))+

xlab("log(Tibia Circumference)")+

ylab("Maximum Growth Marks")+

geom_point() +

scale_y_continuous(breaks = seq(0, 5, by = 1)) +

geom_smooth(method = "glm",

method.args = list(family = "poisson"),

alpha = 0.08, fill = "red",

aes(color = "red")) +

stat_smooth(method = "lm",

fill = "blue", alpha = 0.08,

aes(color = "blue")) +

scale_color_identity(name = "Model fit",

breaks = c("red", "blue"),

labels = c("Poisson", "Linear"),

guide = "legend"); ggp

ggsave("Regressions_MaxGMs_withoutROM.eps", device=cairo_ps, fallback_resolution = 600)

##Femur length and fibula GMs (not logged)

line<-lm(coelo$fibgm~coelo$femlen) #Linear regression of log tibia circumference and tibia growth marks

summary(line)

#Poisson regression, all data

poisson<-glm(coelo$fibgm~coelo$femlen, family = "poisson") #Poisson regression of log tibia circumference and tibia growth marks

summary(poisson)

pseudoR2 <- 1-(poisson$deviance/poisson$null.deviance); pseudoR2 #Pseudo R-squared

pchisq(poisson$deviance, df=poisson$df.residual, lower.tail=FALSE) #Deviance goodness of fit test

#Plot

ggp <- ggplot(data=coelo, aes(femlen, fibgm)) +

theme_bw() +

theme(legend.position = c(0.2,0.7))+

xlab("Femur Length (mm)")+

ylab("Fibula Growth Marks")+

geom_point() +

scale_y_continuous(breaks = seq(0, 5, by = 1)) +

scale_x_continuous(breaks = seq(0, 350, by = 20)) +

geom_smooth(method = "glm",

method.args = list(family = "poisson"),

alpha = 0.08, fill = "red",

aes(color = "red")) +

stat_smooth(method = "lm",

fill = "blue", alpha = 0.08,

aes(color = "blue")) +

scale_color_identity(name = "Model fit",

breaks = c("red", "blue"),

labels = c("Poisson", "Linear"),

guide = "legend"); ggp

ggsave("Regressions_FemurLength_FibGMs_notLogged.eps", device=cairo_ps, fallback_resolution = 600)

##Femur length and fibula GMs (logged)

line<-lm(coelo$fibgm~coelo$logfem) #Linear regression of log tibia circumference and tibia growth marks

summary(line)

#Poisson regression, all data

poisson<-glm(coelo$fibgm~coelo$logfem, family = "poisson") #Poisson regression of log tibia circumference and tibia growth marks

summary(poisson)

pseudoR2 <- 1-(poisson$deviance/poisson$null.deviance); pseudoR2 #Pseudo R-squared

pchisq(poisson$deviance, df=poisson$df.residual, lower.tail=FALSE) #Deviance goodness of fit test

#Plot

ggp <- ggplot(data=coelo, aes(logfem, fibgm)) +

theme_bw() +

theme(legend.position = c(0.2,0.7))+

xlab("log(Femur Length)")+

ylab("Fibula Growth Marks")+

geom_point() +

scale_y_continuous(breaks = seq(0, 5, by = 1)) +

geom_smooth(method = "glm",

method.args = list(family = "poisson"),

alpha = 0.08, fill = "red",

aes(color = "red")) +

stat_smooth(method = "lm",

fill = "blue", alpha = 0.08,

aes(color = "blue")) +

scale_color_identity(name = "Model fit",

breaks = c("red", "blue"),

labels = c("Poisson", "Linear"),

guide = "legend"); ggp

ggsave("Regressions_FemurLength_FibGMs_Logged.eps", device=cairo_ps, fallback_resolution = 600)

##Femur length and maximum GMs (not logged)

line<-lm(coelo$maxgm~coelo$femlen) #Linear regression of log tibia circumference and tibia growth marks

summary(line)

#Poisson regression, all data

poisson<-glm(coelo$maxgm~coelo$femlen, family = "poisson") #Poisson regression of log tibia circumference and tibia growth marks

summary(poisson)

pseudoR2 <- 1-(poisson$deviance/poisson$null.deviance); pseudoR2 #Pseudo R-squared

pchisq(poisson$deviance, df=poisson$df.residual, lower.tail=FALSE) #Deviance goodness of fit test

#Plot

ggp <- ggplot(data=coelo, aes(femlen, maxgm)) +

theme_bw() +

theme(legend.position = c(0.2,0.7))+

xlab("Femur Length (mm)")+

ylab("Maximum Growth Marks")+

geom_point() +

scale_y_continuous(breaks = seq(0, 6, by = 1)) +

geom_smooth(method = "glm",

method.args = list(family = "poisson"),

alpha = 0.08, fill = "red",

aes(color = "red")) +

stat_smooth(method = "lm",

fill = "blue", alpha = 0.08,

aes(color = "blue")) +

scale_color_identity(name = "Model fit",

breaks = c("red", "blue"),

labels = c("Poisson", "Linear"),

guide = "legend"); ggp

ggsave("Regressions_FemurLength_MaxGMs_notLogged.eps", device=cairo_ps, fallback_resolution = 600)

##Femur length and maximum GMs (logged)

line<-lm(coelo$maxgm~coelo$logfem) #Linear regression of log tibia circumference and tibia growth marks

summary(line)

#Poisson regression, all data

poisson<-glm(coelo$maxgm~coelo$logfem, family = "poisson") #Poisson regression of log tibia circumference and tibia growth marks

summary(poisson)

pseudoR2 <- 1-(poisson$deviance/poisson$null.deviance); pseudoR2 #Pseudo R-squared

pchisq(poisson$deviance, df=poisson$df.residual, lower.tail=FALSE) #Deviance goodness of fit test

#Plot

ggp <- ggplot(data=coelo, aes(logfem, maxgm)) +

theme_bw() +

theme(legend.position = c(0.2,0.7))+

xlab("log(Femur Length)")+

ylab("Maximum Growth Marks")+

geom_point() +

scale_y_continuous(breaks = seq(0, 6, by = 1)) +

geom_smooth(method = "glm",

method.args = list(family = "poisson"),

alpha = 0.08, fill = "red",

aes(color = "red")) +

stat_smooth(method = "lm",

fill = "blue", alpha = 0.08,

aes(color = "blue")) +

scale_color_identity(name = "Model fit",

breaks = c("red", "blue"),

labels = c("Poisson", "Linear"),

guide = "legend"); ggp

ggsave("Regressions_FemurLength_MaxGMs_Logged.eps", device=cairo_ps, fallback_resolution = 600)

##### 3D Plots #####

coelo.3d<-coelo[c(1:10, 12:20, 22, 23),] #Remove specimens with missing data (e.g., missing maturity scores)

## 3D plots of the log femur length, maximum growth marks, and maturity score

#3D plot, magma color, lower

scatter3D(coelo.3d$logfem, coelo.3d$maxgm, coelo.3d$matscore.lwr, bty = "u",

col.panel ="lightgray", lwd.grid = .5, type = "h", lwd = 1,

col.grid = "white", xlab = "log(Femur Length [mm])", ylab = "Maximum Growth Marks",

zlab = "Maturity Score (minimum)",

ticktype = "detailed", pch = 16, lty = 3,

cex = 1.3, d = 1, main = "Minimum Maturity Score",

col = magma(256), phi = 20)

##3D plot, magma color, upper

scatter3D(coelo.3d$logfem, coelo.3d$maxgm, coelo.3d$matscore.upr, bty = "u",

col.panel ="lightgray", lwd.grid = .5, type = "h", lwd = 1,

col.grid = "white", xlab = "log(Femur Length [mm])", ylab = "Maximum Growth Marks",

zlab = "Maturity Score (maximum)",

ticktype = "detailed", pch = 16, lty = 3,

cex = 1.3, d = 1, main = "Maximum Maturity Score",

col = magma(256), phi = 20)

##3D plot, magma color, median

scatter3D(coelo.3d$logfem, coelo.3d$maxgm, coelo.3d$matscore.med, bty = "u",

col.panel ="lightgray", lwd.grid = .5, type = "h", lwd = 1,

col.grid = "white", xlab = "log(Femur Length [mm])", ylab = "Maximum Growth Marks",

zlab = "Maturity Score (median)",

ticktype = "detailed", pch = 16, lty = 3,

cex = 1.3, d = 1, main = "Median Maturity Score",

col = magma(256), phi = 20)

## 3D plots of the log tibia circumference, tibia growth marks, and maturity score

coelo.3d.tibia <- coelo[c(1:10,12:20),] #Remove specimens with missing data in these variables

#3D plot, magma color, lower

scatter3D(coelo.3d.tibia$logcir, coelo.3d.tibia$tibgm, coelo.3d.tibia$matscore.lwr, bty = "u",

col.panel ="lightgray", lwd.grid = .5, type = "h", lwd = 1,

col.grid = "white", xlab = "log(Tibia Circumference [mm])", ylab = "Tibia Growth Marks",

zlab = "Maturity Score (minimum)",

ticktype = "detailed", pch = 16, lty = 3,

cex = 1.3, d = 1, main = "Minimum Maturity Score, Tibia",

col = magma(256), phi = 20)

#3D plot, magma color, upper

scatter3D(coelo.3d.tibia$logcir, coelo.3d.tibia$tibgm, coelo.3d.tibia$matscore.upr, bty = "u",

col.panel ="lightgray", lwd.grid = .5, type = "h", lwd = 1,

col.grid = "white", xlab = "log(Tibia Circumference [mm])", ylab = "Tibia Growth Marks",

zlab = "Maturity Score (maximum)",

ticktype = "detailed", pch = 16, lty = 3,

cex = 1.3, d = 1, main = "Maximum Maturity Score, Tibia",

col = magma(256), phi = 20)

#3D plot, magma color, median

scatter3D(coelo.3d.tibia$logcir, coelo.3d.tibia$tibgm, coelo.3d.tibia$matscore.med, bty = "u",

col.panel ="lightgray", lwd.grid = .5, type = "h", lwd = 1,

col.grid = "white", xlab = "log(Tibia Circumference [mm])", ylab = "Tibia Growth Marks",

zlab = "Maturity Score (median)",

ticktype = "detailed", pch = 16, lty = 3,

cex = 1.3, d = 1, main = "Median Maturity Score, Tibia",

col = magma(256), phi = 20)
